# Macroevolutionary patterns in the pelvis, stylopodium and zeugopodium of megalosauroid theropod dinosaurs and their importance for locomotor function

**DOI:** 10.1098/rsos.230481

**Published:** 2023-08-16

**Authors:** Mauro B. S. Lacerda, Jonathas S. Bittencourt, John R. Hutchinson

**Affiliations:** ^1^ Structure and Motion Laboratory, Department of Comparative Biomedical Sciences, The Royal Veterinary College, Hatfield AL9 7TA, UK; ^2^ Pós-Graduação em Zoologia, Instituto de Ciências Biológicas, Universidade Federal de Minas Gerais, Belo Horizonte, 31270-901, Brazil; ^3^ Departamento de Geologia, Instituto de Geociências, Universidade Federal de Minas Gerais, Belo Horizonte 31270-901, Brazil

**Keywords:** disparity, evolution, functional morphology, locomotion, Megalosauroidea, palaeontology

## Abstract

During the Mesozoic, non-avian theropods represented one of the most successful clades globally distributed, with a wide diversity of forms. An example is the clade Megalosauroidea, which included medium- to large-bodied forms. Here, we analyse the macroevolution of the locomotor system in early Theropoda, emphasizing the Megalosauroidea. We scored the *Spinosaurus* neotype in a published taxon-character matrix and described the associated modifications in character states, mapping them onto a phylogeny and using these to study disparity. In the evolution of Megalosauroidea, there was the mosaic emergence of a low swollen ridge; enlargement of the posterior brevis fossa and emergence of a posterodorsal process on the ilium in some megalosauroids; emergence of a femoral head oriented anteromedially and medially angled, and appearance of posterolaterally oriented medial femoral condyles in spinosaurids. The greatest morphological disparity is in the ilium of megalosaurids; the ischium seems to have a high degree of homoplasy; there is a clear distinction in the femoral morphospace regarding megalosauroids and other theropods; piatnitzkysaurids show considerable disparity of zeugopodial characters. These reconstructions of osteological evolution form a stronger basis on which other studies could build, such as mapping of pelvic/appendicular musculature and/or correlating skeletal traits with changes in locomotor function.

## 1. Introduction

During the Jurassic and Cretaceous periods, non-avian theropod dinosaurs represented one of the dominant groups of tetrapods in terrestrial and coastal ecosystems distributed throughout the world. They exhibited a wide diversity and morphological disparity, presenting many body sizes, shape, locomotor and feeding specializations acquired over more than 170 Myr of evolution [1–4]. Throughout the evolutionary history of non-avian theropods, several clades independently radiated, giving rise mostly to medium- to large-sized carnivores (e.g. ceratosaurs, allosauroids, megalosauroids), as well as other clades more specialized in their morphology and ecology (e.g. coelurosaurs), including the only extant clade of theropods, Avialae (or Neornithes) [1,5], which have a great diversity of extant forms and ecological niches.

### 1.1. Megalosauroidea lineage

Megalosauroidea (senior synonym of Spinosauroidea and Torvosauroidea; [6]) was the first major branch of the tetanuran clade to radiate (and, within the clade Orionides, is sister taxon to the lineage leading to Avetheropoda). The probable origin of megalosauroids dates back to the Early Jurassic (Toarcian), with a rapid diversification and dispersion throughout the Jurassic and Early Cretaceous [6–8]. The palaeogeographic distribution of this lineage is broad, with representatives being found on almost all continents/regions, such as South America (e.g. Bonaparte [9]), North America (e.g. Madsen [10]), Africa (e.g. Sereno *et al*. [11]), Europe (e.g. Buckland [12]) and Asia (e.g. Allain *et al*. [13]). Megalosauroids also persisted for a relatively long time-span, between the Middle Jurassic and early Upper Cretaceous [6–8].

Megalosauroidea includes the oldest dinosaur ever described: *Megalosaurus bucklandii* Buckland [12], in addition to *Spinosaurus aegyptiacus* [6,14]. According to Carrano *et al*. [6], Megalosauroidea is a clade including all theropods that are more closely related to *Megalosaurus* than to either *Allosaurus* or *Tyrannosaurus*. Megalosauroids can be diagnosed by features such as extension of the posterior nasal process of the premaxilla, presence of a prominent deltopectoral ridge on the humerus, and an extended anterior maxillary branch; among other synapomorphies [6,7].

In general, megalosauroids represented medium- to large-sized animals, such as *Piatnitzkysaurus* and *Marshosaurus*, from the Jurassic of Patagonia (Argentina) and Morrison Formation (United States), respectively, which could reach 5–6 m in length; megalosaurids, including *Megalosaurus* from the Jurassic of the United Kingdom (varying between 4 and 10 m long); whereas in some spinosaurids, including *Spinosaurus* from the Cretaceous of Africa, the maximum body length has been estimated at between approximately 14 and 17 m [15–18]. This range of body sizes makes Megalosauroidea a useful clade for studying how body size, pelvic appendicular morphology and locomotor function evolved in theropods, but these aspects have been more neglected in the literature than in other clades spanning similar size ranges (mainly allosauroids and coelurosaurs).

According to the most complete phylogeny focusing on Tetanurae, i.e. that of Carrano *et al*. [6] and its modified version by Rauhut *et al*. [8], for example, three main clades compose Megalosauroidea: (i) the earliest-diverging Piatnitzkysauridae (*Piatnitzkysaurus*, *Condorraptor* and *Marshosaurus*), (ii) Megalosauridae (*Megalosaurus*, *Torvosaurus*, *Duriavenator*, *Dubreuillosaurus*, *Afrovenator*, *Piveteasaurus*, *Leshansaurus*, *Magnosaurus*, *Wiehenvenator* and *Poekilopleuron*), and (iii) Spinosauridae (*Spinosaurus*, *Irritator*, *Angaturama*, *Baryonyx* and *Suchomimus*). Within Megalosauroidea, the clade Megalosauria is composed of Megalosauridae + Spinosauridae. Some taxa; e.g. *Monolophosaurus*, *Eustreptospondylus* and *Streptospondylus*; have uncertain phylogenetic placement within Megalosauroidea, as well as *Xuanhanosaurus*, which was recovered as a piatnitzkysaurid by Rauhut *et al*. [8] instead of a metriacanthosaurid as previously [6]. There recently has been an increase in the known biodiversity of Spinosauridae because some new taxa have been described, such as *Ichthyovenator* [13], *Vallibonavenatrix* [19], *Ceratosuchops*, *Riparovenator* [20] and *Iberospinus* [21].

Because of their great diversity and wide geographical distribution, megalosauroids constituted important components of Mesozoic ecosystems, probably partitioning ecological niches with species from related theropod clades, such as predators of Allosauroidea since the Middle Jurassic [22]. Throughout the Mesozoic, taxa considered as ‘top predators’ changed since the Middle Jurassic to the Cenomanian [6,8,14,22–24]. The ecosystems where megalosauroids initially dominated gradually began to be occupied by abelisauroids in Gondwana, as well as several coelurosaur lineages throughout the Upper Jurassic in Laurasia lands [6,22]. Meanwhile, the megalosauroid spinosaurids probably radiated during the Lower Cretaceous, dominating in coastal/riverine ecosystems [16,25,26], then becoming extinct during the Cenomanian, in the Upper Cretaceous [6,22].

The palaeoecological diversification of spinosaurids is evidenced by specializations in cranial morphology, neuroanatomy and tooth morphology [15,27–29], the postcranial skeleton [13,30,31], the pattern of bone microstructure and compactness [15,32], taphonomic [26] and isotopic features suggesting a correlation with coastal palaeoenvironments [25,33]. Furthermore, there is direct evidence of a partially piscivorous diet, with the presence of fish scales and vertebrae found in the gut cavity and dental alveolus of the rostral region of individuals from the clade [17,30], suggesting that megalosauroids inhabited environments near water. Furthermore, Ibrahim *et al*. [15,31] and Fabbri *et al*. [32] proposed that *Spinosaurus* was the first aquatic/amphibious theropod. The purported evidence for the aquatic/amphibious hypothesis includes, among others: (i) ecomorphological adaptations to use the tail for propulsion [31], (ii) increased bone compactness and reduced buoyancy [32], (iii) specializations of the rostrum and much of the skull in spinosaurids, such as the hypertrophied premaxilla and maxilla, and tooth morphology, suggesting a more piscivorous diet or feeding on aquatic prey (e.g. [34–36]; but see also [37–39]), and, by extension, (iv) more than 700 footprints attributed to megalosauroids in Portugal, suggesting that animals of this clade moved to the coast, possibly in search of resources available during low tide [40]. However, the hypothesized aquatic/amphibious and (mainly) piscivorous adaptations of *Spinosaurus* as well as spinosaurids remain highly controversial (e.g. [16,39,41–45]).

This knowledge and lingering questions about non-avian theropods—‘especially megalosauroids'—morphology, locomotor modes, palaeoecology and palaeoenvironments prompts our current study. In this contribution, we analyse the evolution of morphological characters of the theropod locomotor apparatus: the pelvic girdle (i.e. ilium, pubis and ischium) and hindlimb stylopodium and zeugopodium (i.e. femur, tibia and fibula). Initially, to do this, we re-run the phylogenetic analysis of Carrano *et al*. [6] using our modifications of character scorings for spinosaurids. We have four primary aims: (i) evaluate the history of morphological characters of the locomotor system to test whether there are different homoplastic signals in different regions; (ii) reconstruct the ancestral states of the morphology of each trait, searching for correlated macroevolutionary changes of the locomotor apparatus related to hindlimb muscle attachments or other features relevant to locomotion; (iii) where feasible, infer or offer speculations on the possible functional implications of these changes (focusing, in general, on the potential associated musculature), and (iv) test whether different homoplastic signals, based on a morphospace generated by disparity analysis using morphological character states, in different regions of the locomotor apparatus are present in theropods, as well as summarize the most disparate locomotor structures. In conducting these analyses, we also present detailed illustrations and labelled photographs of key character states, which add clarity to our and future phylogenetic analyses, as well as other studies using these data.

#### 1.1.1. Institutional abbreviations

AAOD, Australian Age of Dinosaur Museum of Natural History, Winton, Australia; FMNH, Field Museum of Natural History, Chicago, United States; FSAC, Faculté des Sciences Aïn Chock, University of Casablanca, Casablanca, Morocco; MACN, Museo Argentino de Ciencias Naturales ‘Bernardino Rivadavia’, Buenos Aires, Argentina; MB, Museum für Naturkunde Berlin, Berlin, Germany; MCNA Museo de Ciencias Naturales y Antropológicas (J. C. Moyano) de Mendoza, Mendoza, Argentina; MDS BK Dinosaur Museum, Savannakhet, Laos; MNBH, Musée National de Boubou Hama, Niamey, Niger; MNHNUL (LHNB), Laboratório de História Natural da Batalha, Portugal; MPEF, Museo Paleontológico Egidio Feruglio, Trelew, Argentina; MUCPv, Museo de la Universidad del Comahue, Colección Chocón, Villa El Chocón, Argentina; NHMUK, Natural History Museum, London, United Kingdom; OUMNH, Oxford University Museum of Natural History, Oxford, United Kingdom; PVL, Fundación ‘Miguel Lillo’, San Miguel de Tucumán, Argentina; PW, Paleontological Collections, Department of Mineral Resources, Bangkok, Thailand; SHN, Sociedade de História Natural, Torres Vedras, Portugal; TMM, Texas Vertebrate Paleontology Collections, The University of Texas at Austin, Texas, United States; UCMP, University of California Museum of Paleontology, California, United States; UCRC, University of Chicago Research Collection, Chicago, United States; UMNH, Natural History Museum of Utah, Utah, United States.

## 2. Materials and methods

### 2.1. Anatomical nomenclature

The anatomical terminology of the appendicular skeleton analysed here follows Carrano *et al*. [6]. The nomenclature and homology of the musculoskeletal system follows Hutchinson & Gatesy [46], Hutchinson [47–49] and Carrano & Hutchinson [50]. All taxonomic nomenclature follows Carrano *et al*. [6], with the exception of tetanuran ‘*Dilophosaurus sinensis*', which is currently considered as *Sinosaurus sinensis* [51].

### 2.2. Taxa and character scores: inclusion and analytical procedures

Based on the *Spinosaurus* FSAC-KK 11888 neotype presented by Ibrahim *et al*. [15], the coding of the 68 characters related to the pelvis and stylopodium and zeugopodium detailed in §2.3 below was performed based on the phylogenetic matrix of Carrano *et al*. [6] (see appendices A, B and C). Our morphological study of *Spinosaurus* was performed by accessing the three-dimensional digital model (MorphoSource; UCRC:PV170; ark:/87602/m4/461415) recently made available by Sereno *et al*. [16] and the brief description provided by Ibrahim *et al*. [15]. The scores are shown in table 1 and other modifications are in appendix C. The three-dimensional model of *Suchomimus* (MorphoSource; UCRC:PV171; ark:/87602/m4/486547) provided by Sereno *et al*. [16] was also used in comparative morphology.

**Table 1.** Character state scores for the pelvis, stylopodium and zeugopodium of the *Spinosaurus* neotype—morphological characters 261–328 from [6]. ?—missing data.

Character no.	2222222222	2222222222	2222222222	2222222293	3333333333	3333333333	33333333
	6666666667	7777777778	8888888889	9999999990	0000000001	1111111112	22222222
	1234567890	1234567890	1234567890	1234567890	1234567890	1234567890	12345678
Specimen	Character scores						
FSAC-KK 11888	?001001001	1001110110	02?200?000	0200110?0?	11?01100?0	1101201000	02021101

As there is an ongoing debate whether some isolated material that does not overlap with the original skeleton described by Stromer [52] should be established as *Sp. aegyptiacus* (see, for example, [15,17,31], and arguments by Evers *et al.* [53], Sales & Schultz [54] and Lacerda *et al.* [55]), as well as because our focus here regards the appendicular skeleton related with locomotion, we removed character scores based on isolated rostrum. In our view, the association of the pelvis and hindlimb with *Sp*. *aegyptiacus* [15] is better established, since part of the material described recently was associated with dorsal and cervical vertebrae, as well as with neural spines, which in turn have some overlapping structures with the holotype (see [15,16,52,56]). The pelvic girdle scoring of *Ichthyovenator* (specimens MDS BK10-01–15) included in our analysis was primarily based on the scoring by Rauhut & Pol [57] and the morphological description by Allain *et al*. [13].

Although there are some important updates (e.g. [8,57]), the matrix by Carrano *et al*. [6] was used in our approach because the encoding of postcranial characters is closer to our interpretation of the studied taxa. The matrix analysed is composed of 59 taxa and 352 morphological characters (consisting of 351 taken from Carrano *et al*. [6] and one (no. 352) from Rauhut & Pol [57]). Taxa *Poekilopleuron*, *Streptospondylus* and *Xuanhanosaurus* were excluded because they acted as ‘wildcards’ in a previous analysis [6]. The additional scorings/character modifications in the taxon-character matrix were performed using Mesquite v. 3.6 [58]. As we had changed some character scorings, we felt we needed to re-analyse the phylogeny of our study taxa to see if fundamental relationships were altered. The cladistic analysis was conducted in TNT v. 1.6 [59], following similar parameters adopted by Carrano *et al*. [6] and Hendrickx *et al.* [60]. *Eoraptor* was used as an outgroup to polarize the characters. In our heuristic search, we used the ‘New Technology Search’ algorithms: Sectorial Searches, Ratchet (perturbation phase stopped after 20 substitutions) and Tree-fusing (five rounds), until 100 hits of the same most parsimonious tree (MPT) were achieved using the command xmult = hits 100 rss fuse 5 ratchet 20; a final round of tree bisection reconnection (TBR) branch swapping was performed using the bb command. The characters were analysed under equally weighted parsimony and 32 were considered as ordered, as per [57] (see appendix D). Consistency (CI) and Retention (RI) indices were calculated using the stats.run script in TNT and Bremer support was calculated using suboptimal trees (up to 10 steps). Synapomorphies were mapped using WinClada v. 1.61 [61].

### 2.3. Macroevolutionary history of morphological characters and reconstruction of ancestral states

In order to explore the evolutionary history of morphological characters of the locomotor apparatus in non-avian theropods, with an emphasis on megalosauroids, we used our version of the taxon-character matrix of Carrano *et al*. [6], which focused on tetanuran theropods. Among the 352 morphological characters (see §2.2 and appendices A, B and C), 68 (approx. 19%) are related to the pelvic girdle and hindlimb stylopodium and zeugopodium (character list in appendix B). The characters pertain to the: (i) pelvic elements (character 261); (ii) ilium (characters 262–280); (iii) puboischiadic plate (character 281); (iv) pubis (characters 282–291); (v) ischium (characters 292–300); (vi) femur (characters 301–316); (vii) tibia (characters 317–324), and (viii) fibula (characters 325–328) (appendix B).

Based on the obtained topology, an analysis of the evolution of each of the aforementioned morphological characters of the locomotor apparatus was performed using Mesquite software [58]. Through the maximum-likelihood method for character state reconstruction (i.e. mapping or tracing), which calculates the ancestral state that maximizes the probability of the observed states to have evolved under a stochastic model [62], the state of each character in the most recent common ancestor (MRCA) of each theropod clade was also estimated; however, focusing our analysis on megalosauroids. The evolutionary model assumed in the MRCA reconstructions was Mk1 (Markov k-state 1), which considers the rate of character state changes as equally probable, since this method denotes greater effectiveness when compared with the parsimony criterion [62]; however, when polymorphic characters were present (characters: 281, 284, 292, 301, 306, 325), the MRCA reconstructions were made using the parsimony criterion instead of likelihood calculations. Using the likelihood parameter, the proportional likelihood (pl) of each estimated MRCA is given in % values [58].

### 2.4. Disparity analyses

We calculated six Euclidean distance matrices based on the modified version of the taxon-character matrix from Carrano *et al*. [6], using the aforementioned morphological characters (§2.1, appendix B). Each of the new matrices separately represented the elements of the locomotor apparatus studied here: (i) all characters of the pelvic girdle and hindlimb stylopodium and zeugopodium (characters 261–328); (ii) ilium (characters 262–280); (iii) pubis (characters 282–291); (iv) ischium (characters 292–300); (v) femur (characters 301–316), and (vi) tibia/fibula (characters 317–328), which were divided to assess the influence of each of these structures on the disparity analyses (explained below).

We then removed taxa that do not have the studied pelvic and hindlimb elements preserved. Analysis (i) was conducted leaving 52 operational taxonomic units (OTUs); analysis (ii) 50 OTUs; analysis (iii) 48 OTUs; analysis (iv) 43 OTUs; analysis (v) and (vi) 46 OTUs (see details in appendix E). All polymorphic characters were treated as unknown (?) in these analyses.

Subsequently, using the PAST v. 4.01 program [63], the data were sorted based on the Euclidean dissimilarity index for each of the six matrices through principal coordinate analysis (PCO), in order to visualize the disparity of the morphological characters based on the distribution of the taxa in a two-dimensional morphospace of reduced dimensionality. PCO, when compared with principal component analysis (PCA), is more suitable for disparity analysis of phylogenetic characters, as it better handles missing data, which are common in palaeontological studies, and are more appropriate with distance/dissimilarity matrices [64,65]. Although there is a wide debate about corrections and adaptations to disparity methods that use discrete character states from phylogenetic matrices, as well as continuous data (e.g. [64,66]), in this work, we order the data in a way similar to other studies (e.g. [67]) that used the Euclidean distances among taxa posteriorly ordinated in a morphospace. All datasets used in this work can be downloaded in [68].

## 3. Results and discussion

### 3.1. Cladistic analysis

**Figure 1.**
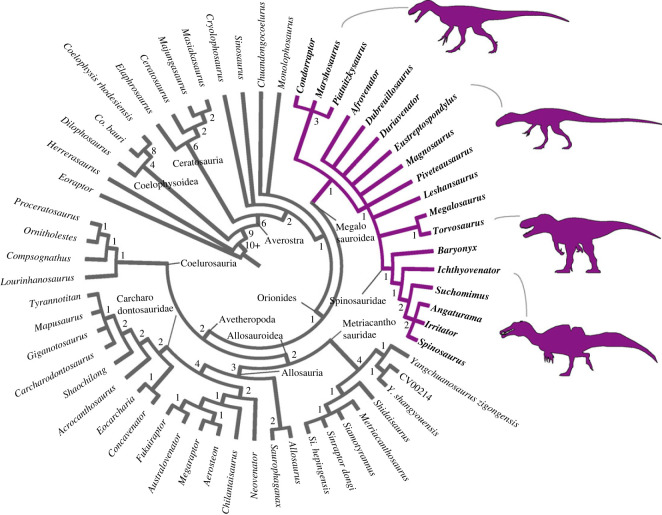
Results of phylogenetic analysis. Strict consensus cladogram of the most parsimonious trees retrieved (tree length = 1067; CI = 0.407; RI = 0.677), Bremer support is displayed below each node. The highlighted and illustrated clade represents Megalosauroidea. Silhouettes were downloaded from phylopic.org; see Acknowledgements.

In the first round of analysis, 428 MPTs were reached with 1067 steps each. Subsequent TBR application of the retained trees resulted in a total of 1800 MPTs each of 1067 steps. The strict consensus of these trees reached is shown in cladogram in figure 1. Based on the consensus topology, the homoplastic index CI and RI were 0.407 and 0.677, respectively.

In general, the topology obtained is congruent with the results of Carrano *et al*. [6], because it recovers successive clades within Theropoda such as Coelophysoidea, Ceratosauria and the larger Tetanurae clade composed of successive taxa at its base (e.g. *Sinosaurus*, *Cryolophosaurus*) as successive sister lineages of the clade Orionides, which is composed of Megalosauroidea and the larger clade Avetheropoda (figure 1). Unlike a recent study [8], our analysis recovered *Dilophosaurus* within Coelophysoidea and *Monolophosaurus* as an early Tetanurae, in agreement with [6].

Although the large theropod clades obtained are compatible with Carrano *et al*.'s [6] results, some clades internally lost resolution with the inclusion of the spinosaurid *Ichthyovenator* and our changes to the characters of *Spinosaurus*. In our analysis, the Megalosauroidea clade is composed of two main clades: the early diverging clade Piatnitzkysauridae; which includes *Piatnitzkysaurus*, *Marshosaurus* and *Condorraptor* (unresolved internal relationships); and a later-diverging clade Spinosauridae, which includes *Baryonyx*, *Ichthyovenator*, *Suchomimus* and Spinosaurinae (*Spinosaurus*, *Irritator* and *Angaturama*) (figure 1).

The classic Megalosauridae, which includes *Eustreptospondylus* as the early diverging species, followed by the Megalosaurinae (*Duriavenator*, *Megalosaurus* and *Torvosaurus*) and Afrovenatorinae (*Afrovenator*, *Magnosaurus*, *Dubreuillosaurus*, *Leshansaurus* and *Piveteasaurus*) clades [6] were not recovered (however, see further discussions in §4.1). Our analysis retrieved a large unresolved polytomy where the clade internally recovered is comparable to Megalosaurinae (*Megalosaurus* + *Torvosaurus*) (figure 1).

Regarding Spinosauridae, *Ichthyovenator* was recovered as a non-spinosaurine spinosaurid as previously proposed [13,57]. Indeed, in our analysis the Baryonychinae clade was not recovered, with *Baryonyx*, *Ichthyovenator* and *Suchomimus* as a sequence of successive lineages, the latter being the outgroup of Spinosaurinae. Finally, although Spinosaurinae was recovered as a clade, similar to results obtained by Carrano *et al*. [6], in our phylogeny the internal relationships remain unresolved (figure 1). Although megalosaurids were recovered without resolution, for ease of comparison, here we presume megalosaurid monophyly (*sensu* [6]).

### 3.2. Evolutionary history and ancestral states of theropod pelvic girdle and hindlimb stylopodium and zeugopodium characters

#### 3.2.1. Pelvic elements

The articulations of the pubis, ilium and ischium (character 261) in theropods can be unfused (261[0]) or fused (261[1]) in adults. The plesiomorphic condition is observed in the sauropodomorph *Eoraptor* and most other theropods, with exceptions in coelophysoids and ceratosaurs such as *Ceratosaurus* and *Masiakasaurus*, in which the pelvic elements are fused (pelvic fusion also occurs convergently within Avialae). Throughout non-avian tetanuran evolution, the plesiomorphic condition generally persisted, including in all megalosauroids mapped. The reconstruction of this character state for the MRCA of megalosauroids suggests that it had unfused pelvic elements (pl = 99%), unlike the condition in coelophysoids, whose MRCA had a fused pelvic girdle (pl = 97%). This fusion might have biomechanical importance for the overall strength of the pelvis, but this remains untested; finite-element analyses could give insights into the potential consequences.

#### 3.2.2. Ilium

Nineteen of the studied characters (27.94%) are related to the ilium. The ilium represents a structure on which a substantial part of the locomotor musculature originates/originated in theropods [46,48–50,69–77], both from the dorsal and ventral groups (e.g. *M*. *iliotibialis*, *M*. *ilotrochantericus caudalis*, *M*. *flexor tibialis externus*, *M*. *caudofemoralis brevis*).

The pneumatic foramen and the internal cavities of the ilium (character 262) of theropods may be absent (262[0]) or present (262[1]). Most of the studied species retain the plesiomorphic condition; changes are rare (*sensu* [62]). This pneumatization is present in carcharodontosaurs; specifically in neovenatorids; so the MRCA of neovenatorids had a pneumatic foramen and internal cavities in the ilium (pl = 98%). The pneumatization presumably indicates expanded posterior (abdominal) air sacs (e.g. [78,79]), although whether this had important functional ramifications is unclear.

**Figure 2.**
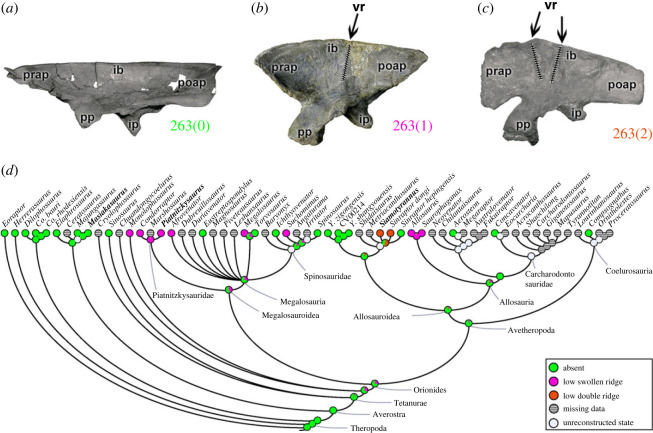
Evolutionary history of character 263 (ilium, vertical ridge on lateral surface of blade dorsal to acetabulum) and the ancestral state reconstruction. Illustration of the left ilium in lateral view: (*a*) *Masiakasaurus* FMNH PR 2485; (*b*) *Piatnitzkysaurus* MACN-Pv-CH 895; (*c*) *Siamotyrannus* PW 9-1. (*d*) Phylogenetic tree of Tetanurae showing the reconstruction of ancestral character state for each node. (*a*) modified from [80] and (*c*) modified from [6]. Not to scale. ib, iliac blade; ip, ischiadic peduncle; poap, postacetabular process; pp, pubic peduncle; prap, preacetabular process; vr, vertical ridge.

A vertical ridge may be present on the lateral surface of the ilium, dorsal to the acetabulum (character 263). This crest is absent (263[0]) in *Eoraptor* and early theropods (e.g. *Masiakasaurus*; figure 2*a*). A low and robust vertical ridge (263[1]) is present in early tetanurans such as *Monolophosaurus*, piatnitzkysaurids (e.g. *Piatnitzkysaurus*; figure 2*b*) and allosaurids. A vertical ridge that represents a low and double structure (263[2]) is present only in some allosauroids, specifically in metriacanthosaurines (*Siamotyrannus* (figure 2*c*) and *Sinraptor*). In megalosauroids, the absence of the iliac ridge is noted in *Eustreptospondylus* and *Torvosaurus*, as well as in the spinosaurids *Ichthyovenator* and *Spinosaurus*. Meanwhile, a low swollen ridge is present in *Megalosaurus*, *Afrovenator*, *Suchomimus* and the early diverging piatnitzkysaurids. The MRCA of megalosauroids probably lacked the vertical ridge in the ilium (pl = 58%); however, the MRCA of piatnitzkysaurids probably had a robust ridge (pl = 93%) (figure 2*d*). This ridge(s) is thought to potentially indicate clearer separation between the origins of *M*. *ilotrochantericus caudalis* (ITC)/*M*. *iliofemoralis externus* and *M*. *iliofibularis* (e.g. [50]).

**Figure 3.**
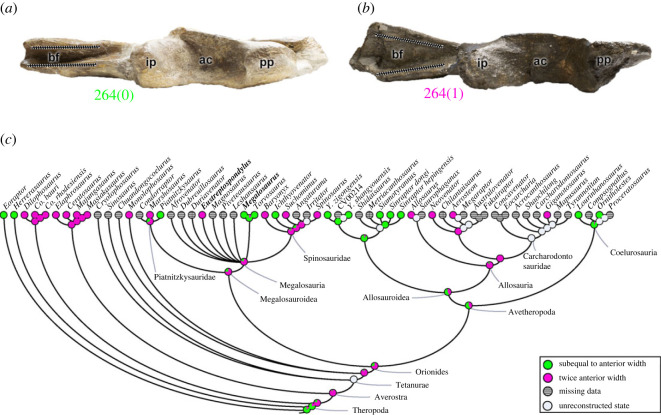
Evolutionary history of character 264 (ilium, posterior width of brevis fossa) and the ancestral state reconstruction. Illustration of the right ilium in ventral view: (*a*) *Megalosaurus* OUMNH J.13560; (*b*) *Eustreptospondylus* OUMNH J.13558/E01. (*c*) Phylogenetic tree of Tetanurae showing the reconstruction of ancestral character state for each node. Not to scale. ac, acetabulum; bf, brevis fossa; ip, ischiadic peduncle; pp, pubic peduncle.

The brevis or postacetabular fossa is present in the posteromedial portion of the ilium in theropods and other dinosaurs. The posterior width of this cavity (character 264), may be approximately equal to the anterior width, with subparallel margins (264[0]), or it may have its posterior width twice the anterior width; i.e. enlarged posteriorly (264[1]) (figure 3). The plesiomorphic condition exists in *Eoraptor* and *Herrerasaurus* and persists in the megalosauroids *Piatnitzkysaurus*, *Megalosaurus* (figure 3*a*), *Torvosaurus* and *Ichthyovenator*, but also in metriacanthosaurids and some coelurosaurs. Yet some early diverging clades as coelophysoids and ceratosaurs, as well as allosaurs, have the brevis fossa enlarged posteriorly (264[1]). The character state in the MRCA of megalosauroids suggests the presence of a posteriorly wide brevis fossa (pl = 75%), based on the morphology of the piatnitzkysaurid *Marshosaurus*, as well as *Eustreptospondylus* (figure 3*b*), *Baryonyx*, *Suchomimus* and *Spinosaurus*. The same applies to both MRCAs of piatnitzkysaurids (pl = 68%) and spinosaurids (pl = 87%) (figure 3*c*). The brevis fossa is related to the origin of the *M*. *caudofemoralis brevis* (CFB), similar to that in Crocodylia, and inferred for Theropoda and other dinosaurs in general [48,50,69,70,72–74,76,81], the posterior enlargement of this fossa may indicate greater size of the CFB muscle.

**Figure 4.**
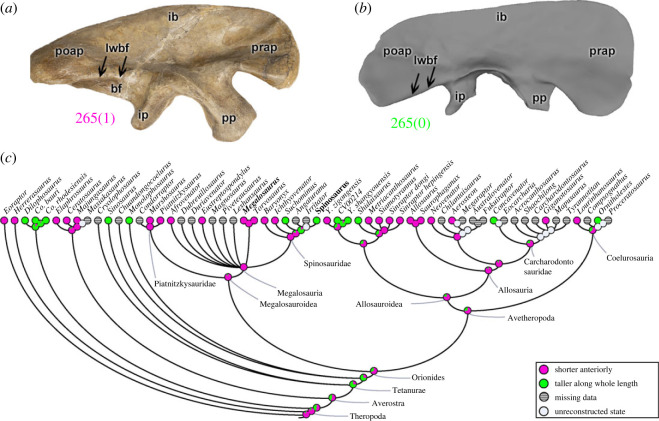
Evolutionary history of character 265 (ilium, height of lateral wall of brevis fossa relative to medial wall) and the ancestral state reconstruction. Illustration of the right ilium in lateral view: (*a*) *Megalosaurus* OUMNH J.13560; (*b*) *Spinosaurus* FSAC-KK 11888. (*c*) Phylogenetic tree of Tetanurae showing the reconstruction of ancestral character state for each node. (*b*) Based on the three-dimensional digital model provided by Sereno *et al.* [16]. Not to scale. bf, brevis fossa; lwbf, lateral wall of brevis fossa; ib, iliac blade; ip, ischiadic peduncle; poap, postacetabular process; pp, pubic peduncle; prap, preacetabular process.

By comparing the delimitations of the brevis fossa, it is evident that the height of the lateral wall relative to the medial wall (character 265) can be greater along the entire length of the brevis fossa (265[0]), as noted in coelophysoids, early diverging tetanurans, and some avetheropods (i.e. *Shidaisaurus*, *Yangchuanosaurus*, *Concavenator* and *Ornitholestes*). A distinct condition is evident when the most anterior portion of the lateral wall is short, exposing part of the medial wall in lateral view (265[1]); this condition is widely mapped across the phylogeny, including *Eoraptor*, *Herrerasaurus* and many other theropods (figure 4). In megalosauroids, the ancestral condition (even if mapped as state ‘1’); i.e. the boundaries are shorter anteriorly; is present in several taxa (e.g. *Megalosaurus*; figure 4*a*), except *Suchomimus* and *Spinosaurus* (figure 4*b*). The MRCA reconstruction in megalosauroids indicates that the lateral wall was anteriorly shorter (pl = 92%) (figure 4*c*). Some of the morphological variations of the brevis fossa observed within megalosauroids may indicate changes of the boundaries of the CFB origin in some taxa; for example, *Megalosaurus* and *Torvosaurus* have a narrower brevis fossa than in *Eustreptospondylus* and *Spinosaurus*, and thus might have had a more restricted CFB origin and smaller muscle size.

**Figure 5.**
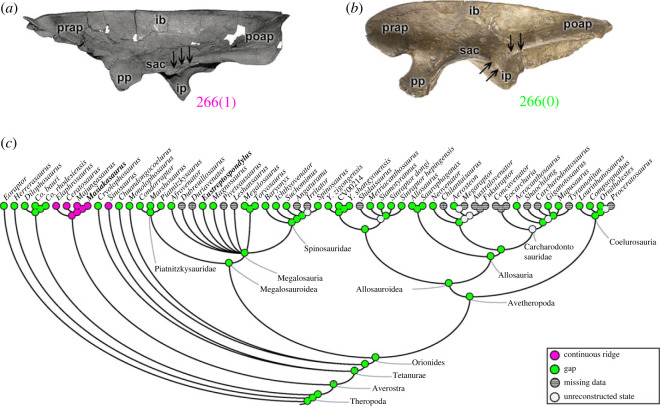
Evolutionary history of character 266 (ilium, morphology between supraacetabular crest and brevis shelf on lateral surface) and the ancestral state reconstruction. Illustration of the left ilium in lateral view: (*a*) *Masiakasaurus* FMNH PR 2485; (*b*) *Eustreptospondylus* OUMNH J.13558/E01 (mirrored). (*c*) Phylogenetic tree of Tetanurae showing the reconstruction of ancestral character state for each node. (*a*) Modified from [80]. Not to scale. ib, iliac blade; ip, ischiadic peduncle; poap, postacetabular process; pp, pubic peduncle; prap, preacetabular process; sac, supraacetabular crest.

**Figure 6.**
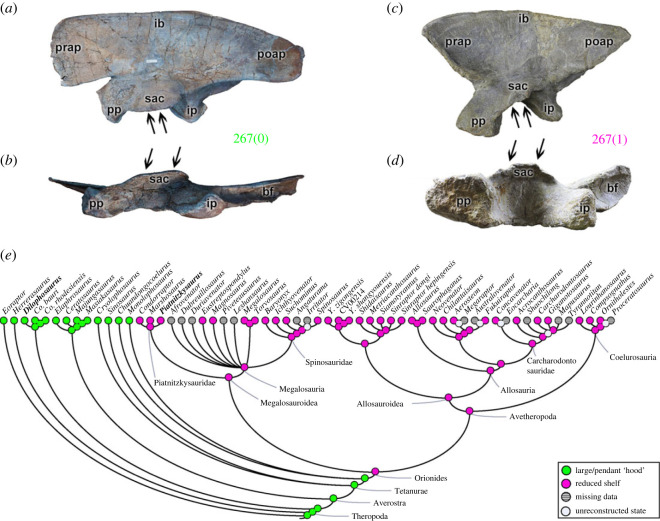
Evolutionary history of character 267 (ilium, ventrolateral development of supraacetabular crest) and the ancestral state reconstruction. Illustration of the left ilium in lateral and ventral views: (*a*,*b*) *Dilophosaurus* TMM 43646-1; (*c*,*d*) *Piatnitzkysaurus* MACN-Pv-CH 895. (*e*) Phylogenetic tree of Tetanurae showing the reconstruction of ancestral character state for each node. (*a*,*c*) Lateral view, (*b*) and (*d*) ventral view. (*a*,*b*) Modified from [82]. Not to scale. bf, brevis fossa; ib, iliac blade; ip, ischiadic peduncle; poap, postacetabular process; pp, pubic peduncle; prap, preacetabular process; sac, supraacetabular crest.

The posterolateral portion of the ilium, between the supraacetabular crest and the brevis shelf (character 266), may have a ‘gap’ (266[0]), as observed in most theropods (e.g. *Eustreptospondylus*; figure 5*b*). A continuous ridge (266[1]) is a morphological acquisition of ceratosaurs (pl = 97% for the MRCA) (e.g. *Masiakasaurus*; figure 5*a*), and independently in the early tetanuran *Sinosaurus* (figure 5*c*). The development of the supraacetabular ridge of the ilium in its ventrolateral portion (character 267) may culminate in a large/suspended, or ‘hood’-shaped structure (267[0]), as in *Eoraptor* and non-Orionides theropods (coelophysoids (e.g. *Dilophosaurus*; figure 6*a*,*b*), ceratosaurs and early tetanurans). The supraacetabular crest of the ilium with a reduced projection (267[1]) (e.g. *Piatnitzkysaurus*; figure 6*c*,*d*) is a rare change that is observed in Orionides (pl = 98% for the MRCA) (figure 6*e*). The ventrolateral region between the supraacetabular crest and the brevis shelf may also indicate a boundary of the CFB origin (e.g. [70]), and, based on our analysis, it represents a structure with evolutionary stability in megalosauroids. Differences between theropods in the various states of characters related to the brevis fossa may indicate interspecific variation in where the CFB originated, or even how large it was overall.

**Figure 7.**
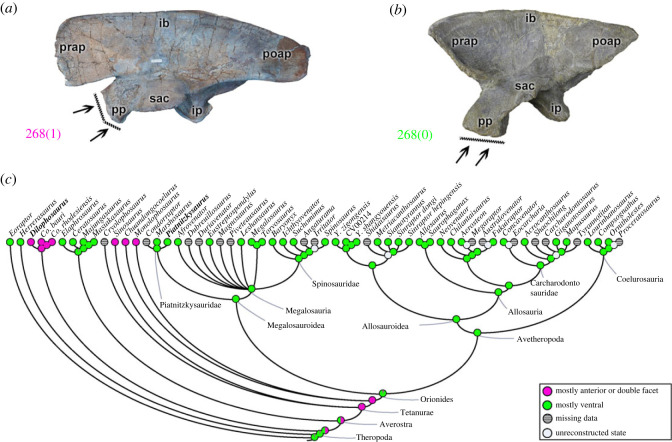
Evolutionary history of character 268 (ilium, orientation of pubic peduncle) and the ancestral state reconstruction. Illustration of the left ilium in lateral view: (*a*) *Dilophosaurus* TMM 43646-1; (*b*) *Piatnitzkysaurus* MACN-Pv-CH 895. (*c*) Phylogenetic tree of Tetanurae showing the reconstruction of ancestral character state for each node. (*a*) Modified from [82]. Not to scale. ib, iliac blade; ip, ischiadic peduncle; poap, postacetabular process; pp, pubic peduncle; prap, preacetabular process; sac, supraacetabular crest.

The orientation of the pubic peduncle in the anteroventral portion of the ilium (character 268) can be mainly in a ventral direction (268[0]), as in most theropods (e.g. *Piatnitzkysaurus*; figure 7*b*); or in a more anteriorly located peduncle with a ‘double facet’ projecting anteriorly and ventrally (268[1]), as present in coelophysoids (including *Dilophosaurus*; figure 7*a*) and the early diverging tetanurans (figure 7*c*). Based on this, the reconstruction of the ancestral character state for the MRCA of Tetanurae suggests the apomorphic condition (pl = 96%); however, in the reconstruction for the MRCA of Orionides the ancestral orientation of the pubic peduncle was more ventral (pl = 99%) and conservative within megalosauroids. The functional implications of these orientations are obscure.

**Figure 8.**
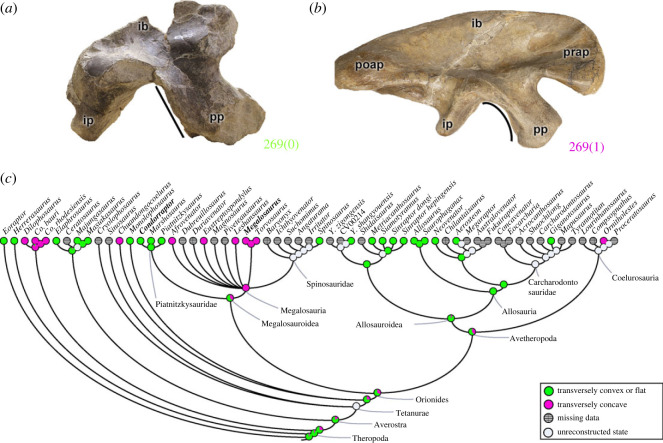
Evolutionary history of character 269 (ilium, shape of acetabular margin of pubic peduncle) and the ancestral state reconstruction. Illustration of the left ilium in lateral view: (*a*) *Condorraptor* MPEF-PV 1687 (mirrored); (*b*) *Megalosaurus* OUMNH J.13560. (*c*) Phylogenetic tree of Tetanurae showing the reconstruction of ancestral character state for each node. Not to scale. ib, iliac blade; ip, ischiadic peduncle; poap, postacetabular process; pp, pubic peduncle; prap, preacetabular process.

The pubic peduncle has its posterior margin delimited by the acetabulum. The shape of this margin (character 269) can be transversely flat or convex (269[0]), as in *Eoraptor*, *Herrerasaurus*, ceratosaurs and avetheropods, in addition to piatnitzkysaurids (e.g. *Condorraptor*; figure 8*a*) and *Spinosaurus*. A transversely concave posterior margin of the pubic peduncle (269[1]) is evident in coelophysoids, megalosaurids (e.g. *Megalosaurus*; figure 8*b*), as well as independently in *Chuandongocoelurus* and *Ornitholestes* (figure 8*c*). Considering that piatnitzkysaurids and *Spinosaurus* have the shape of the pubic peduncle transversely flat or convex, the MRCA of megalosauroids probably had the plesiomorphic condition (pl = 63%). Again, it is unclear if this variation had important functional relevance.

The relative sizes of the iliac articulations, the pubic and ischial joints (character 270), can be approximately equal (270[0]), as in *Eoraptor* and non-Orionides theropods. However, in the clade composed of *Chuandongocoelurus* and *Monolophosaurus* at the base and all Orionides (pl = 99% for the MRCA), the pubic joint is more robust, being greater than or equal to 130% of the ischial articulation (270[1]). In addition to this proportionality, the morphology of the ischial peduncle (character 271) is rounded (271[0]) in the majority of theropods, being evolutionary stable. The exception is an abbreviated distalmost ischial peduncle (271[1]), which is present in the carcharodontosaurid *Concavenator*, and the coelurosaurs *Compsognathus* and *Ornitholestes*. These traits might relate to relative differences in the strengths or mobility of these joints between pelvic elements, but it is pure speculation if this is the case or if the differences would be of biologically relevant magnitudes.

The ratio between the width and length of the pubic peduncle (character 272) represents a multi-state character in theropods. This ratio is less than or equal to 1 (272[0]) as observed in non-Orionides theropods, and independently in the megalosauroids *Eustreptospondylus*, *Suchomimus* and *Spinosaurus*. A ratio between 1.3 and 1.75 (272[1]) is prevalent in tetanurans (except in *Sinosaurus* and the aforementioned megalosauroids), in metriacanthosaurids (except *Siamotyrannus*) and independently in the coelurosaur *Ornitholestes*. In allosaurs, *Siamotyrannus* and *Ornitholestes*, the ratio is greater than 2 (272[2]). Although *Eustreptospondylus*, *Suchomimus* and *Spinosaurus* have the plesiomorphic condition, the MRCA of megalosauroids had a ratio between 1.3 and 1.75 between the width and length of the pubic peduncle (pl = 99%). The pubic peduncle region may indicate the ventral boundary of the origin of *M*. *puboischiofemoralis internus 1* (PIFI1) (e.g. [50,70]), so a longer peduncle might correlate with an expanded PIFI1 origin, or a partial shift of that origin from medial (as in Crocodylia) to lateral (as in Aves). As suggested by our analysis, only the proportionality of this structure relative to the ischial peduncle presents variation in megalosauroids; mainly internally in megalosaurids and spinosaurids.

In the medial region of the ilium, the portion adjacent to the preacetabular notch or ‘cuppedicus fossa’ (related to the origin of PIFI1 and/or PIFI2; e.g. [46,48,75]) (character 273), may be smooth, without a crest (273[0]), as in all non-avetheropod taxa (except the coelophysoid *Coelophysis rhodesiensis* and the megalosauroid *Ichthyovenator*) and independently in *Sinraptor dongi*. In avetheropods, except *Si*. *dongi*, there is a crest in the portion adjacent to the ‘cuppedicus fossa’ (273[1]); which is also the condition in *Co*. *rhodesiensis* and *Ichthyovenator*. In neovenatorids this crest is not only present but also is well developed, forming a projection (273[2]). With the exception of *Ichthyovenator*, the plesiomorphic condition is predominant in megalosauroids and in the MRCA (pl = 99%). The development of this fossa is widely held to relate to the shift of the PIFI1 and PIFI2 from the medial pelvis and preacetabular vertebrae to the lateral iliac surface across the lineage to Aves (see references above), with consequences for the sizes and moment arms of those muscles (e.g. [83,84]).

**Figure 9.**
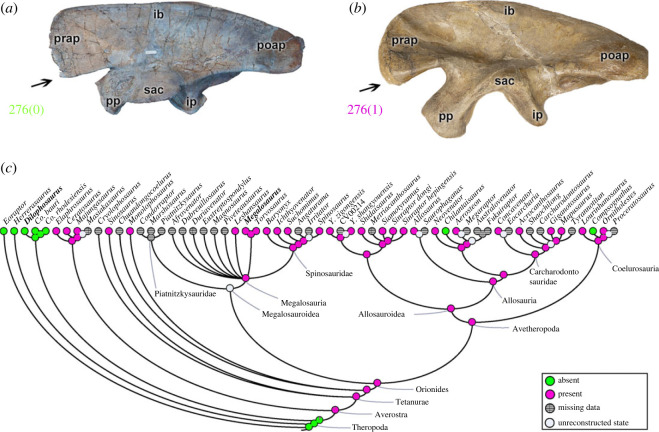
Evolutionary history of character 276 (ilium, anteroventral lobe of preacetabular process) and the ancestral state reconstruction. Illustration of the left ilium in lateral view: (*a*) *Dilophosaurus* TMM 43646-1; (*b*) *Megalosaurus* OUMNH J.13560 (mirrored). (*c*) Phylogenetic tree of Tetanurae showing the reconstruction of ancestral character state for each node. (*a*) Modified from [82]. Not to scale. ib, iliac blade; ip, ischiadic peduncle; poap, postacetabular process; pp, pubic peduncle; prap, preacetabular process; sac, supraacetabular crest.

The length of the preacetabular process in relation to the anterior edge of the pubic peduncle (character 274) was an evolutionarily stable feature in theropods. A preacetabular process with an anterior part of the pubic peduncle that projects anteriorly at the same point (274[0]) is evident only in *Eoraptor* and *Herrerasaurus*. The preacetabular process projects anteriorly well beyond the pubic peduncle (274[1]) in Neotheropoda (pl = 98% for the MRCA). The same macroevolutionary pattern exists for the depth of the preacetabular process (character 275), which is shallow (275[0]) in *Eoraptor* and *Herrerasaurus*, and is deep in Neotheropoda (275[1]) (pl = 98% for the MRCA). The most anteroventral region of the preacetabular process (character 276) may have its edges without any lobed structure (276[0]) as observed in non-Averostra theropods (e.g. *Dilophosaurus*; figure 9*a*), *Chilantaisaurus* and *Compsognathus* (figure 9*c*). Other than these exceptions, averostran taxa have an anteroventral lobe of the preacetabular process (276[1]) (e.g. *Megalosaurus*; figure 9*b*). All mapped megalosauroids have the apomorphic condition; however, the MRCA of megalosauroids cannot be reconstructed, as none of the piatnitzkysaurids have the anteriormost part of the ilium preserved. Derived states of characters 274–276 probably correlate with anterior expansion of the ITC muscle origin across Theropoda. The preacetabular process of the ilium is associated with the origin of at least three muscles of the dorsal hindlimb group: *M*. *iliotibialis 1* (IT1) on the lateral dorsal/anterior rim, ITC on the anteriormost lateral surface, and PIFI1 or PIFI2 muscle(s) on the preacetabular region, as noted above (e.g. [46,48–50,72–74]). Based on the character history, there was some conservatism in the morphology of these muscle origins within Megalosauroidea.

**Figure 10.**
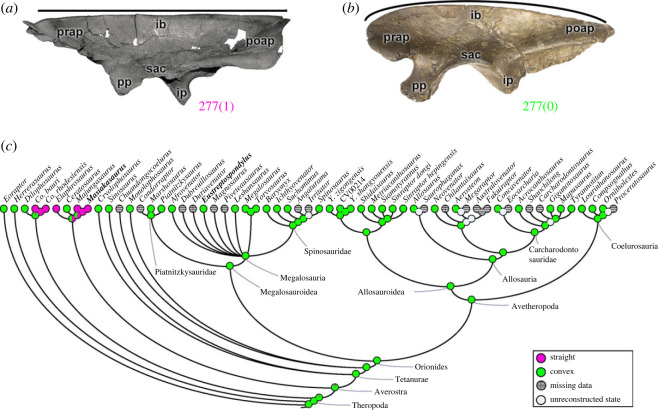
Evolutionary history of character 277 (ilium, shape of dorsal margin) and the ancestral state reconstruction. Illustration of the left ilium in lateral view: (*a*) *Masiakasaurus* FMNH PR 2485; (*b*) *Eustreptospondylus* OUMNH J.13558/E01 (mirrored). (*c*) Phylogenetic tree of Tetanurae showing the reconstruction of ancestral character state for each node. (*a*) Modified from [80]. Not to scale. ib, iliac blade, ip, ischiadic peduncle; poap, postacetabular process; pp, pubic peduncle; prap, preacetabular process; sac, supraacetabular crest.

The shape of the dorsal border of the ilium (character 277) in lateromedial view may have a convex margin (277[0]), as in most theropods including all Orionides clades (e.g. *Eustreptospondylus*; figure 10*b*), with the exception of a straight dorsal margin morphology (277[1]), which is in coelophysids and ceratosaurs (except *Ceratosaurus*) (e.g. *Masiakasaurus*; figure 10*a*). Interestingly, because of the plesiomorphic (state 0) character status in *Ceratosaurus*, the reconstruction of the MRCA of ceratosaurs is ambiguous (pl = 49% of state 0; and pl = 51% of state 1) (figure 10*c*). At least two hindlimb muscles of the dorsal group have their origin from the dorsal border of the ilium other than the preacetabular process: *Mm*. *iliotibiales 2* and *3* (IT2, IT3; e.g. [48,73]). This character would relate in some, perhaps minor, way to the extents and sizes of these muscles.

The ratio of the width of the postacetabular process of the ilium relative to the ischial peduncle (character 278) may be less than or equal to 1 (278[0]), as per the condition in *Eoraptor* and *Herrerasaurus*. The morphology of remaining neotheropods analysed exhibits a ratio greater than 1 (278[1]) (pl = 98% for the MRCA of neotheropods). The same macroevolutionary pattern pertains to the depth of the postacetabular process (character 279), being shallow (279[0]) in *Eoraptor* and *Herrerasaurus*, whereas in neotheropods this process is deep (279[1]) (pl = 99% on the MRCA of neotheropods). The derived state would be expected to correlate with expansion of all postacetabular iliac muscle origins (and likely sizes), to some degree; as per below.

**Figure 11.**
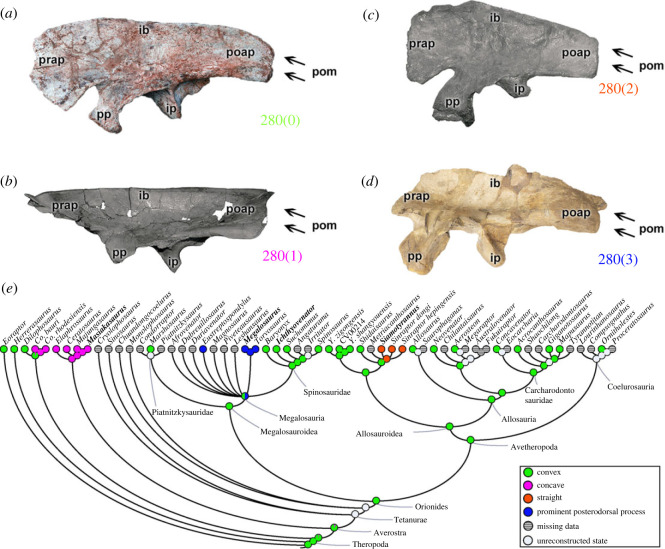
Evolutionary history of character 280 (ilium, shape of posterior margin of postacetabular process) and the ancestral state reconstruction. Illustration of the left ilium in lateral view: (*a*) *Ichthyovenator* MDS BK 10-09; (*b*) *Masiakasaurus* FMNH PR 2485; (*c*) *Siamotyrannus* PW 9-1; (*d*) *Megalosaurus* OUMNH J.29882 (mirrored). (*e*) Phylogenetic tree of Tetanurae showing the reconstruction of ancestral character state for each node. (*a*) Modified from [13], (*b*) modified from [80] and (*c*) modified from [6]. Not to scale. ib, iliac blade; ip, ischiadic peduncle; poap, postacetabular process; pom, posterior margin; pp, pubic peduncle; prap, preacetabular process.

One of the major morphological variations observed in the ilium of early theropods is related to the shape of the posterior margin of the postacetabular process (character 280) (figure 11). A convex posterior margin (280[0]) is in several taxa such as *Eoraptor*, *Dilophosaurus*, the megalosauroid *Marshosaurus* and spinosaurids (e.g. *Ichthyovenator*; figure 11*a*), in addition to avetheropods (except some Metriacanthosaurinae). A concave posterior margin of the postacetabular process (280[1]) is a synapomorphy of ceratosaurs (e.g. *Masiakasaurus*; figure 11*b*); homoplastically present in coelophysids. A straight posterior margin (280[2]) exists only in the metriacanthosaurines *Siamotyrannus* (figure 11*c*) and *Sinraptor*. By contrast, a prominent postacetabular process in the dorsal region, but with the absence of the posteroventral process (280[3]), is evident only in megalosaurids such as *Eustreptospondylus*, *Megalosaurus* (figure 11*d*) and *Torvosaurus*. However, due to the plesiomorphic state in *Marshosaurus*, the reconstruction for the megalosauroid MRCA indicates a convex posterior margin of the ilium (pl = 98%) (figure 11*e*). The postacetabular iliac region should have held the origins of the *M*. *flexor tibialis externus* (FTE) and *M*. *iliofibularis* (ILFB), as well as part of the posterior delimitation of the IT3 [48–50,74,83]; based on this, the FTE, ILFB and IT3 muscles probably varied in size/position within megalosauroids.

#### 3.2.3. Puboischiadic plate

The morphology and presence/absence of foramina and notches in the puboischiadic plate (see [48]), which is the region ventral to the acetabulum (character 281), varies across Theropoda. A medially completely fused plate, with the presence of three foramina (281[0]) is present in *Eoraptor*, *Herrerasaurus*, coelophysoids (although it is polymorphic in *Dilophosaurus* ((281[0,1]); see [6]), in the early diverging ceratosaur *Elaphrosaurus*, and non-Orionides tetanurans, as well as megalosauroids (except *Afrovenator* and *Leshansaurus*). Notably, the tetanuran *Sinosaurus* and *Baryonyx* have intraspecific/intraindividual variations in this character (281[0,1]). A puboischiadic plate opening medially, with only the obturator foramen of the pubis, which has 1–2 notches (281[1]), is found in the ceratosaurs *Ceratosaurus* and *Masiakasaurus*, as well as in the metriacanthosaurids *Yangchuanosaurus*. This character state also is polymorphic (281[1,2]) in the megalosaurid *Leshansaurus*, the metriacanthosaurid *Yangchuanosaurus* (CV00214) and *Si*. *hepingensis*, and the carcharodontosaurid *Mapusaurus*. A reinterpretation of this morphological character (e.g. with an ontogenetic perspective, if it indeed varied across ontogeny) could improve coding in taxa where the character is mapped as plesiomorphic (i.e. *Dilophosaurus*, *Sinosaurus*, *Leshansaurus*, *Baryonyx* and *Mapusaurus*). A puboischiadic plate that is medially open, without any fenestrae, but with 1–2 notches (281[2]), is present, in addition to the aforementioned taxa, in *Afrovenator* and other avetheropods; with the exceptions noted above. Even with some variations and derived states within megalosauroids, the most parsimonious MRCA reconstruction indicates a fully closed puboischiadic plate. The puboischiadic plate region in early theropods, such as *Staurikosaurus* [69] and *Coelophysis* [70] should have provided an origin for the *M*. *puboischiofemoralis externus 3* (PIFE3), that plesiomorphically extended anteriorly from the ischium, ventral to the acetabulum. By contrast, for example, in the carcharodontosaurid *Acrocanthosaurus* [71] and in later coelurosaurs [50], the origin of the PIFE3 muscle seems to have been more restricted, posterior to the puboischiadic plate. In some maniraptoran taxa, the origin is positioned further ventrally on the ischium [81]. These changes correlate with what may be reduction of this muscle's size (if the origin is indicative of that; e.g. [85]) but later shifting of the origin to the medial puboischiadic membrane on the lineage to Aves (and perhaps re-expansion of the muscle).

#### 3.2.4. Pubis

Among the analysed characters, 10 of them relate to the pubis (14.7%). In Crocodylia, Aves and non-avian theropods, the pubis is the origin of *M*. *ambiens* (AMB) of the dorsal group, as well as PIFE1 and PIFE2 of the ventral group [49,50,69,70,72,73].

**Figure 12.**
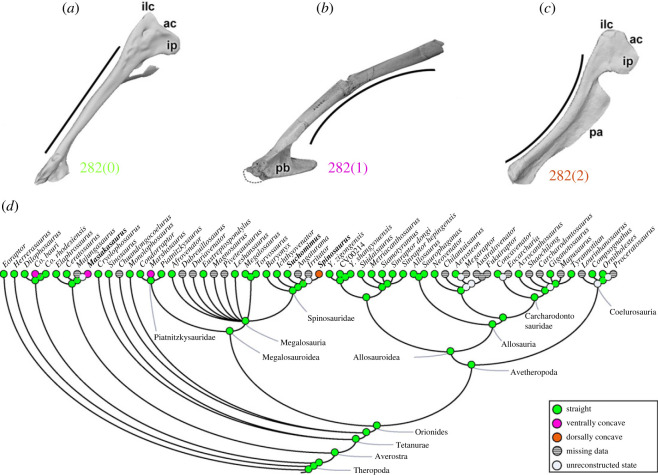
Evolutionary history of character 282 (pubis, shaft orientation) and the ancestral state reconstruction. Illustration of the left pubis in lateral view: (*a*) *Suchomimus* MNBH GAD500; (*b*) *Masiakasaurus* FMNH PR 2470; (*c*) *Spinosaurus* FSAC-KK 11888. (*d*) Phylogenetic tree of Tetanurae showing the reconstruction of ancestral character state for each node. (*a*,*c*) Based on the three-dimensional digital model provided by Sereno *et al.* [16] and (*b*) modified from [80]. Not to scale. ac, acetabulum; ilc, iliac contact; ip, ischiadic peduncle; pa, pubic apron; pb, pubic boot.

Generally in theropods, the orientation of the main axis of the pubis (character 282) presents a conservative morphological condition: a straight-shafted pubis (282[0]), in almost all analysed species including early theropods (e.g. *Suchomimus*; figure 12*a*). There are a few exceptions where the pubic orientation is ventrally concave (282[1]), a trait that evolved independently in *Co*. *bauri*, *Masiakasaurus* (figure 12*b*) and the megalosauroid *Marshosaurus*. An autapomorphic feature is notable in *Spinosaurus*, which has a dorsally concave shaft of the pubis (282[2]) (figure 12*c*). Even with derived states in *Marshosaurus* and *Spinosaurus*, the MRCA reconstruction of megalosauroids indicates a straight-shafted pubis (pl = 99%) (figure 12*d*). It is conceivable that different orientations of the pubic shaft might have altered the PIFE1 and PIFE2 muscles' moment arms [84].

The pubic apical articulation (character 283) can be unfused in adult individuals (283[0]), as in early theropods (except ceratosaurs) and early tetanurans including megalosauroids (except *Afrovenator*), whereas in avetheropods only *Si*. *hepingensis* has this state. A fused apical articulation (283[1]) is evident in ceratosaurs, the megalosaurid *Afrovenator* and allosauroids. Despite the apomorphic feature in *Afrovenator*, the state in the MRCA of megalosauroids has the unfused condition (pl = 95%).

The distal pubis (character 284); proximal to any fusion or distalmost contact; may have a gap between the right and left pubes (284[0]), as noted in non-averostran theropods; or have them in contact (284[1]), as unambiguously seen in *Ceratosaurus*. However, this is a polymorphic character (possibly with ontogenetic variation) in the ceratosaur *Elaphrosaurus*, in the tetanuran *Monolophosaurus*, in the megalosauroids *Megalosaurus*, *Torvosaurus* and *Suchomimus*, as well as in the avetheropods *Shidaisaurus*, *Yangchuanosaurus zigongensis* and *Saurophaganax*. Even with the aforementioned variations, the predominant condition in theropods is with pubes contacting distally, but this contact forms a proximal–distal ‘gap’ called the interpubic fenestra (284[2]) in most of the averostran species. An interpubic fenestra is predominant in *Marshosaurus*, *Piatnitzkysaurus*, *Afrovenator*, *Baryonyx*, *Ichthyovenator* and *Spinosaurus*; and is the most parsimonious condition reconstructed for the MRCA of megalosauroids.

The distal pubis is well known to present a structure called the pubic ‘boot’ (i.e. posterior projection of the distal portion of the bone; e.g. [48]). The angle between the main axis of the pubis and the pubic boot (character 285) can vary between 75° and 90° (285[0]), as in most theropod species. The one exception, where the angle is less than 60° (285[1]), represents a synapomorphy of metriacanthosaurine allosauroids [6]. However, the state of this character is poorly characterized in early theropods such as coelophysoids and megalosauroids. The morphology of the pubic symphysis (character 286) independently observed in the early theropod *Herrerasaurus* and spinosaurids (except *Baryonyx*) represents a marginal structure (286[0]); however, all other analysed theropods have a wider pubic symphysis (286[1]); which is also the reconstructed state in the MRCA of megalosauroids (pl = 99%). As with other characters related to pelvic fusion, characters 283–285 might relate to increased rigidity or strength of the pelvis, and the boot may have provided stronger resistance to supporting body weight during sitting (as well as abdominal muscle insertions and inspiratory flow; see [86]).

A pubic obturator foramen (character 287) as a small subcircular structure (287[0]) is present in almost all non-avetheropods; however, the predominant condition in avetheropods is the presence of a large, oval foramen (287[1]), including also the non-avetheropod megalosauroids *Ichthyovenator* and *Suchomimus*. It is unclear what these ventral pelvic foramina might indicate in terms of soft tissues in early theropods, but the general trend across Theropoda is the appearance of these foramina and then their expansion, breaking up the formerly united puboischiadic plate (e.g. [48]). This reduction of the ventral pelvic surface area is probably related to reduced muscle sizes (e.g. PIFE3) or even losses of muscles (e.g. parts of the *flexor cruris* ventral group).

**Figure 13.**
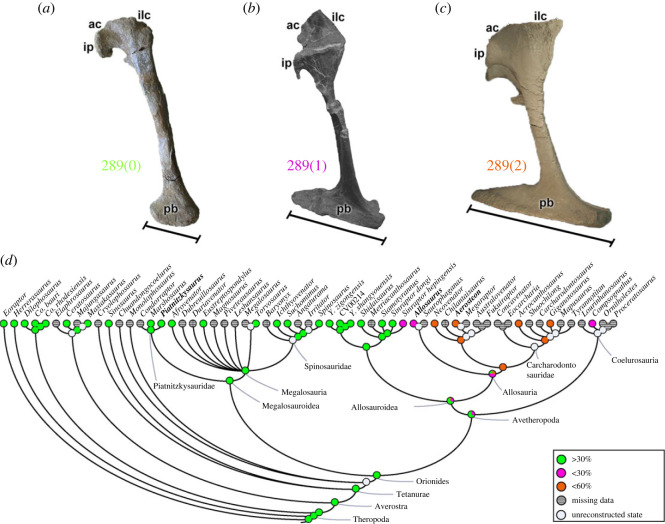
Evolutionary history of character 289 (pubis, boot length relative to shaft length) and the ancestral state reconstruction. Illustration of the left pubis in lateral view: (*a*) *Piatnitzkysaurus* PVL 4073; (*b*) *Allosaurus* MNHNUL/AND.001/007; (*c*) *Aerosteon* MCNA-PV-3137 (cast; mirrored). (*d*) Phylogenetic tree of Tetanurae showing the reconstruction of ancestral character state for each node. (*b*) Modified from [87] and (*c*) modified from [78]. Not to scale. ac, acetabulum; ilc, iliac contact; ip, ischiadic peduncle; pb, pubic boot.

An expansion of the anterodistal-most part of the pubis (character 288) may be absent (288[0]), as in non-allosaur theropods. Contrastingly, this expansion is present in the allosaur clade (288[1]). The maximum length of the pubic boot in relation to the length of the shaft (character 289) can be less than 30% (289[0]), as in non-avetheropod theropods (e.g. *Piatnitzkysaurus*; figure 13*a*) and metriacanthosaurids (except *Si*. *hepingensis*); or greater than 30% (289[1]) in the metriacanthosaurid *Si*. *hepingensis*, the allosaurid *Allosaurus* (figure 13*b*) and the coelurosaur Compsognathus. A pubis expansion greater than 60% (289[2]) is present in carcharodontosaurs (e.g. *Aerosteon*; figure 13*c*). The shape of the pubic boot from a ventral view (character 290) may be triangular (290[0]), as in most theropods analysed, except in *Herrerasaurus* and late coelurosaur *Compsognathus*, whose pubic boot is narrow with subparallel margins (290[1]). It would be interesting to ascertain if the size of this structure relates to increases of body sizes in tetanuran theropods, in light of its potential role in static weight-bearing.

The shape of the iliopubic articulation (character 291) largely is conserved in theropods, being planoconcave (291[0]) in all non-ceratosaur theropods analysed. Yet the ceratosaurs *Ceratosaurus*, *Majungasaurus* and *Masiakasaurus* have an iliopubic articulation in a ball and socket form (291[1]).

#### 3.2.5. Ischium

The taxon-character matrix analysed here involves nine morphological characters (13.2%) related to the ischium. In the ischium of Crocodylia and non-avian theropods, some ventral hindlimb muscle groups originate(d) here, such as *M*. *flexor tibialis internus 1*,*3* (FTI1 and FTI3), in addition to the PIFE3 muscle and *M*. *adductor femoris* (ADD1 and ADD2) (e.g. [46,49,50,72,73]).

A ratio of the length of the ischium to the pubis (character 292) between 75% and 80% (292[0]), independently evolved in *Eoraptor*, *Herrerasaurus*, *Co*. *bauri*, *Masiakasaurus*, *Monolophosaurus* and megalosauroids (except *Torvosaurus* and *Spinosaurus*). A smaller ratio, less than or equal to 70% (292[1]), is shared between the coelophysoids *Dilophosaurus*, *Co*. *rhodesiensis*, *Ceratosaurus* (though polymorphic) and coelurosaurs *Ornitholestes* and *Compsognathus*. Ischia with a larger ratio, greater than 80% (292[2]), exist in *Sinosaurus*, *Torvosaurus*, *Spinosaurus* and allosauroids (except *Neovenator*). The most parsimonious reconstruction for the MRCA of megalosauroids suggests an ischium length relative to pubis length between 75% and 80% (state 0). The derived state's ratio seems to be achieved by a lengthening of the pubis; perhaps with the contribution of the enlargement of the pubic boot.

**Figure 14.**
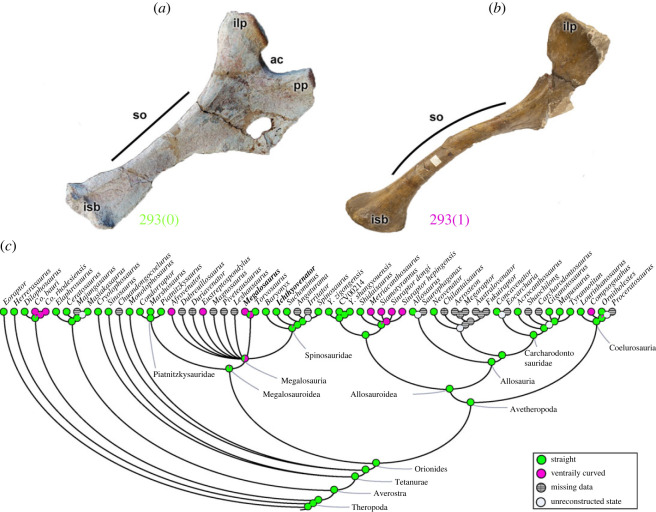
Evolutionary history of character 293 (ischium, shaft orientation) and the ancestral state reconstruction. Illustration of the left ischium in lateral view: (*a*) *Ichthyovenator* MDS BK 10–13; (*b*) *Megalosaurus* OUMNH J.13565 (mirrored). (*c*) Phylogenetic tree of Tetanurae showing the reconstruction of ancestral character state for each node. (*a*) Modified from [13]. Not to scale. ac, acetabulum; ilp, iliac peduncle; isb, ischial boot; pp, pubic peduncle; so, shaft orientation.

The orientation of the main axis of the ischium (character 293) is straight (293[0]) in *Eoraptor* and several analysed theropods (e.g. *Ichthyovenator*; figure 14*a*). A ventrally curved main ischial axis (293[1]) evolved repeatedly in some theropod clades such as coelophysids, the megalosaurids *Afrovenator*, *Eustreptospondylus*, *Megalosaurus* (figure 14*b*), metriacanthosaurine allosauroids and *Compsognathus*. Although the megalosauroid MRCA probably had a straight ischium (pl = 93%), at least three aforementioned megalosaurids had the derived condition (i.e. ventrally curved ischial shaft) (figure 14*c*). These shape differences would at least slightly alter the moment arms of muscles with ischial origins (e.g. [84]).

The ilioischiadic articulation (character 294) has two forms in theropods: a concave plane (294[0]), present in most clades, the exceptions being *Co*. *rhodesiensis*, *Majungasaurus* and *Masiakasaurus*, *Ichthyovenator*, and Carcharodontosauridae, which have a ball and socket articulation (294[1]). Although the spinosaurid *Ichthyovenator* presents the apomorphic condition, the MRCA of megalosauroids had a planoconcave articulation (pl = 99%).

The ischial antitrochanter (character 295) is a large, notch-shaped structure (295[0]) in non-tetanuran theropods and in *Ichthyovenator*. In all other taxa, the ischial antitrochanter is reduced (295[1]), which probably was the state in the MRCA of Megalosauroidea (pl = 99%). The functional significance of various acetabular ‘antitrochanter’ structures around the ilium and ischium remains unclear, but is thought to relate to differences in hip joint function, and deserves deeper investigation (see [88]).

**Figure 15.**
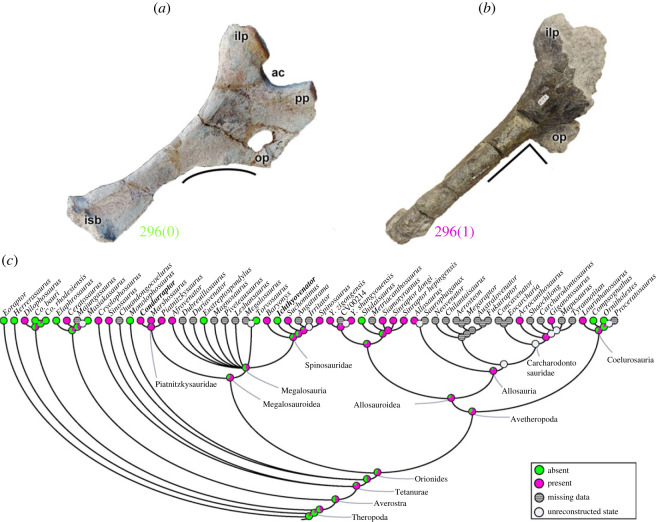
Evolutionary history of character 296 (ischium, notch ventral to obturator process) and the ancestral state reconstruction. Illustration of the left ischium in lateral view: (*a*) *Ichthyovenator* MDS BK 10–13; (*b*) *Condorraptor* MPEF-PV 1689 (mirrored). (*c*) Phylogenetic tree of Tetanurae showing the reconstruction of ancestral character state for each node. (*a*) Modified from [13]. Not to scale. ac, acetabulum; ilp, iliac peduncle; isb, ischial boot; pp, pubic peduncle; op, obturator process.

A notch (character 296) ventral to the ischial obturator process may be absent (296[0]), as in many theropods including *Herrerasaurus* and some tetanurans (e.g. *Ichthyovenator*; figure 15*a*). This notch is present (296[1]) in *Dilophosaurus* and *Ceratosaurus*, and unites Tetanurae (pl = 79%) (e.g. *Condorraptor*; figure 15*b*). Three megalosauroids (*Eustreptospondylus*, *Torvosaurus* and *Ichthyovenator* (figure 15*a*)) have a reversal of this character, but the MRCA of megalosauroids probably had the apomorphic state (296[1]; pl = 66%) (figure 15*c*). The PIFE3 and ADD1 muscle origins associated with this ischial region (e.g. [49,50,69,72]) may be reduced in size in taxa with the derived state.

An unexpanded ischial symphysis (297[0]) exists in *Eoraptor* and several theropod clades: coelophysids, ceratosaurs (except *Masiakasaurus*), early tetanurans, megalosauroids (except *Marshosaurus*, megalosaurids and *Ichthyovenator*), as well as allosaurs. An ischial symphysis expanded as an apron (297[1]) appeared independently in *Masiakasaurus* and the allosaur clade. Additionally, the state is variable in megalosauroids because *Marshosaurus*, megalosaurids and *Ichthyovenator* have the derived condition (i.e. expanded ischial symphysis). An unexpanded ischial symphysis is a plausible condition for the megalosauroid MRCA (pl = 72%); however, the MRCA of spinosaurids have an ambiguous reconstruction (pl = ∼50% for each state). An apron-like expansion could correlate with enlarged muscle origins such as for ADD1 and PIFE3.

The cross-sectional shape of the middle axes of the articulated ischia (character 298) commonly is oval (298[0]) in theropods. In some clades such as coelophysids and metriacanthosaurids, however, the cross-section is heart-shaped, with the medial portions protruding posteriorly (298[1]).

**Figure 16.**
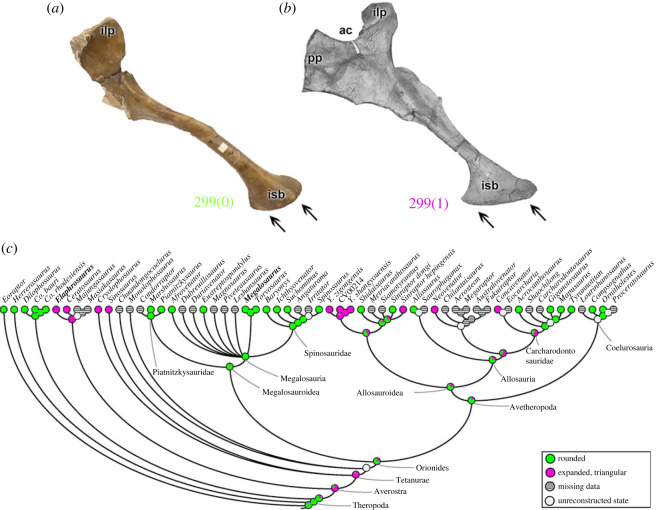
Evolutionary history of character 299 (ischium, morphology of distal end) and the ancestral state reconstruction. Illustration of the left ischium in lateral view: (*a*) *Megalosaurus* OUMNH J.13565; (*b*) *Elaphrosaurus* MB R 4960. (*c*) Phylogenetic tree of Tetanurae showing the reconstruction of ancestral character state for each node. (*b*) Modified from [89]. Not to scale. ac, acetabulum; ilp, iliac peduncle; isb, ischial boot; pp, pubic peduncle.

The distal portion of the ischium (character 299) has a rounded tip (299[0]) in most of the analysed theropods (e.g. *Megalosaurus*; figure 16*a*). An ischium with a triangularly expanded distal end (299[1]) is observed in ceratosaurs (e.g. *Elaphrosaurus*; figure 16*b*), *Cryolophosaurus* and *Sinosaurus*, *Yangchuanosaurus*, *Si*. *hepingensis*, *Neovenator* and *Concavenator*. Ischia with rounded ends are probable for the MRCA of Neotheropoda (pl = 85%); however, the averostran MRCA seemingly was triangular (pl = 81%). Finally, the Megalosauroidea MRCA had the plesiomorphic condition (pl = 95%) that remained conservative within the clade (figure 16*c*). As with the pubic boot (see above), this expansion must relate in some obscure way to static weight support as well as abdominal/caudal muscle attachments.

The distalmost ischia (character 300), may remain unfused in adult individuals (300[0]) as in most theropods. Distal ischial fusion in adults (300[1]) occurs in *Dilophosaurus*, ceratosaurs, early tetanurans and *Sinosaurus*, in metriacanthosaurids (except *Si*. *hepingensis*) and *Neovenator*. Again, this fusion-related trait might vary ontogenetically.

#### 3.2.6. Stylopodium (femur)

Sixteen morphological characters (23.5%) in this study relate to the femur. Several of the muscles that originate from the pelvic girdle and (post cervical) vertebrae insert on the femur. In Crocodylia and non-avian theropods (as well as Aves), the proximal region of the femur is the insertion site of the PIFI1-2, PIFE1-3, ITC and *iliofemoralis externus* (IFE) muscles or their homologues [47,49,50,69,70,72–75]. A large portion of the posterior diaphysis of the femur has the *Mm*. *caudofemorales* (CFB and CFL) attached and, distally, the *Mm*. *adductores femores 1* and *2* (ADD1 and ADD2). However, the femoral diaphysis also predominantly is/was the area of origin of some dorsal *triceps femoris* group muscles. Furthermore, the metaphyseal region is the origin for several lower limb muscles (e.g. *M*. *gastrocnemius externus*/*lateralis* (GE); *M*. *flexor halluces longus* (FHL); *M*. *extensor digitorum longus* (EDL)) that ultimately inserted onto the tarsals, metatarsus or phalanges and unguals [49,50,70,90].

**Figure 17.**
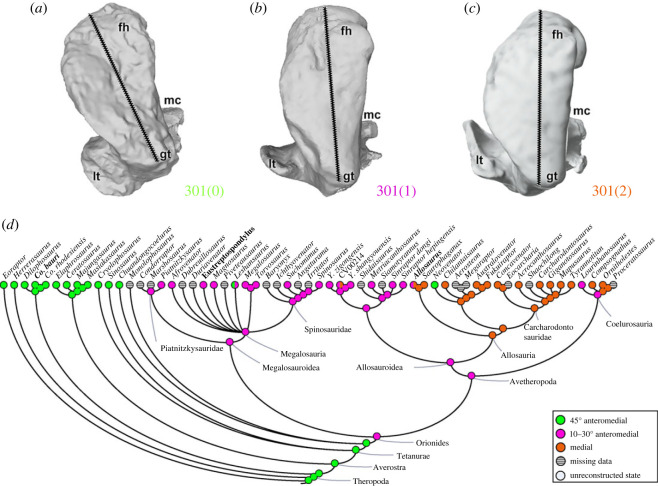
Evolutionary history of character 301 (femur, head orientation) and the ancestral state reconstruction. Illustration of the left femur in proximal view: (*a*) *Coelophysis* UCMP 129618 (mirrored); (*b*) *Eustreptospondylus* OUMNH J.13558/F02; (*c*) *Allosaurus* UMNH VP 7892. (*d*) Phylogenetic tree of Tetanurae showing the reconstruction of ancestral character state for each node. (*a*) Based on the three-dimensional digital model provided by University of California Museum of Paleontology (MorphoSource; UCMP:V:129618) and (*c*) based on the three-dimensional digital model provided by Natural History Museum of Utah (MorphoSource; UMNHVP:7892; ark:/87602/m4/509051). Not to scale. fh, femoral head; gt, greater trochanter; lt, lesser trochanter; mc, medial condyle.

The femoral head's orientation varies in two ways, more anteriorly and medially versus more dorsoventrally and medially, so it is scored as two characters. The femoral head is oriented (character 301) 45° anteromedially (301[0]) in most early theropods including coelophysoids (e.g. *Co*. *bauri*; figure 17*a*) and ceratosaurs. An intermediate condition, which varies between 10° and 30° anteromedially (301[1]), is the morphological feature in megalosauroids (e.g. *Eustreptospondylus*; figure 17*b*), metriacanthosaurids and the coelurosaur *Lourinhanosaurus*. A medially oriented femoral head (301[2]) is a synapomorphy of allosauroids (e.g. *Allosaurus*; figure 17*c*), and independently evolved in *Compsognathus* and *Ornitholestes*. Notably, two states (301[0,1]) exist in the megalosaurid *Leshansaurus* and in *Allosaurus* (301[1,2]). An intermediate condition (i.e. femoral head orientation between 10° and 30°) is the most parsimonious state for the megalosauroid MRCA (figure 17*d*). A more medially oriented femoral head should have contributed to a more parasagittal gait and support (e.g. [46,83,91,92]).

**Figure 18.**
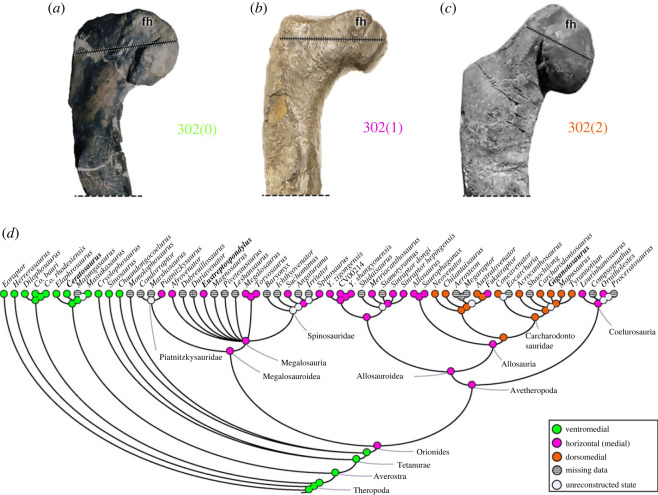
Evolutionary history of character 302 (femur, head angle) and the ancestral state reconstruction. Illustration of the left femur in posterior view: (*a*) *Ceratosaurus* UMNH 5278; (*b*) *Eustreptospondylus* OUMNH J.13558/F02; (*c*) *Giganotosaurus* MUCPv-Ch 1 (mirrored). (*d*) Phylogenetic tree of Tetanurae showing the reconstruction of ancestral character state for each node. (*c*) Modified from [93]. Not to scale. fh, femoral head.

The orientation of the femoral head (character 302) is ventromedial (302[0]), again in most early theropods (e.g. *Ceratosaurus*; figure 18*a*). As a transitional state, however, the head of the femur is horizontal (medial) (302[1]) in megalosauroids (e.g. *Eustreptospondylus*; figure 18*b*), metriacanthosaurids, allosaurids, in the neovenatorid *Fukuiraptor* and coelurosaurs; a feature evolved in the MRCA of Orionides (pl = 97%). A dorsomedially inclined femoral head (302[2]) is a feature widespread in carcharodontosaurs (e.g. *Giganotosaurus*; figure 18*c*), except *Fukuiraptor*. These differences in orientation should have implications for biomechanical loading of the proximal femur (e.g. [71,94]).

An articular groove (or fovea capitis, e.g. [95,96]) that is oriented obliquely along the main axis of the femoral head's proximal surface (character 303) may be absent (303[0]) in theropods as it is in the non-theropod *Eoraptor* and all analysed avetheropods; or present (303[1]) as in many early theropods including megalosauroids. The groove of the oblique ligament on the surface of the posterior portion of the femoral head (character 304) is shallow, with its bordering lip not projecting beyond the posterior surface of the femoral head (304[0]) only in megalosauroids, specifically the *Afrovenator*, *Megalosaurus*, *Torvosaurus* and *Spinosaurus*. Although this character is unknown in *Eoraptor* and *Herrerasaurus*, all neotheropods (MRCA pl = 99%) studied have a deep groove of the oblique ligament (considered as derived feature [6]), with medial delimitation by the posterior lip of the well-developed groove (304[1]). Although the aforementioned megalosauroids have a shallow groove, the MRCA is reconstructed with the derived condition (state 1; pl = 98%), suggesting a reversion within this clade.

**Figure 19.**
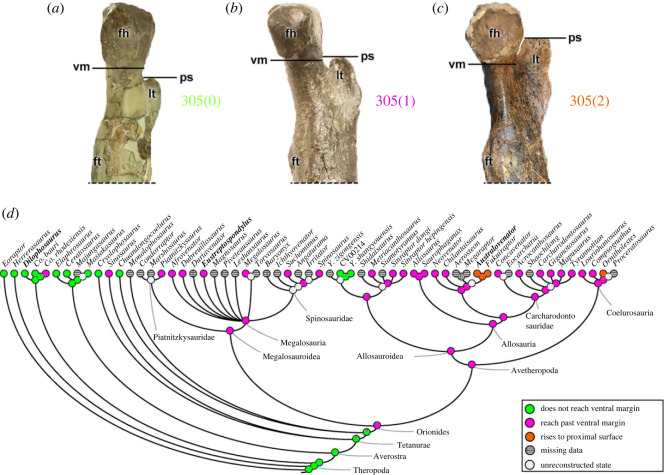
Evolutionary history of character 305 (femur, placement of lesser trochanter relative to femoral head) and the ancestral state reconstruction. Illustration of the left femur in medial view: (*a*) *Dilophosaurus* UCMP 37302; (*b*) *Eustreptospondylus* OUMNH J.13558/F02; (*c*) *Australovenator* AODF604 (mirrored). (*d*) Phylogenetic tree of Tetanurae showing the reconstruction of ancestral character state for each node. (*a*) Modified from [82] and (*c*) modified from [97]. Not to scale. fh, femoral head; ft, fourth trochanter; lt, lesser trochanter; ps, proximal surface; vm, ventral margin.

The position of the lesser trochanter (also called anterior trochanter) in relation to the femoral head (character 305) does not reach the ventral/distal margin (305[0]), as in early theropods (except *Co*. *rhodesiensis* and *Sinosaurus*), and the metriacanthosaurid *Yangchuanosaurus* (*Y*. *shangyouensis* + CV00214) (also *Dilophosaurus*; figure 19*a*). In most studied theropods of the Orionides clade, however, the lesser trochanter projects proximal to the ventral margin of the femoral head (305[1]), including megalosauroids (e.g. *Eustreptospondylus*; figure 19*b*) and averostrans. The carcharodontosaurs *Australovenator* (figure 19*c*) and *Fukuiraptor*, and the coelurosaur *Ornitholestes*, on the other hand, have a lesser trochanter reaching the proximal surface of the femoral head (305[2]). Although a more distally restricted lesser trochanter relative to the femoral head is widely seen in early theropods, a lesser trochanter projecting beyond the ventral margin of the femoral head is the plausible condition for the MRCA of Orionides (pl = 94%) (figure 19*d*).

The morphology of the insertion sites of the anterolateral muscles of the thigh, on the proximal portion of the femur (character 306; e.g. [47]) may represent a continuous lesser trochanter shelf (306[0]), as in *Eoraptor* and *Herrerasaurus*, as well as polymorphic in coelophysoids and *Ceratosaurus* (306[0,1]). Other theropod species, including all Tetanurae (see [47]), except the early diverging *Sinosaurus* and the ceratosaurs *Majungasaurus* and *Masiakasaurus*, have a distinct insertion site of the lesser trochanter (discrete rugosity) (306[1]) on the proximal region of the femur. The most parsimonious condition in MRCA of megalosauroids is a distinct lesser trochanter and trochanteric shelf reduced to a bulge (state 1). These features probably relate to the *ischiotrochantericus* (ISTR), IFE and ITC muscles (e.g. [47,69,70,98]), with the ITC moving proximally and anteriorly with the lesser trochanter (altering its moment arms; [84]), while the IFE and ISTR maintained conservative positions.

A predominant theropod (and other diapsid reptile) feature is the presence of a fourth trochanter of the femur (character 307), which is the attachment site of the powerful CFL and CFB musculature [47,50,72,76,99]. A fourth trochanter as a laterally prominent semioval projection (307[0]) is the predominant condition in theropods. Exceptions are shown by the unnamed allosauroid (CV00214) and *Chilantaisaurus*, as well as by the coelurosaurs *Compsognathus* and *Ornitholestes*, which have a fourth trochanter that is poorly developed or even absent (307[1]). Gatesy [76] outlined how reduction of the fourth trochanter (and tail, and CFL muscle) indicates a gradual shift in locomotor function from more hip-driven to more knee-driven across the lineage to Aves.

**Figure 20.**
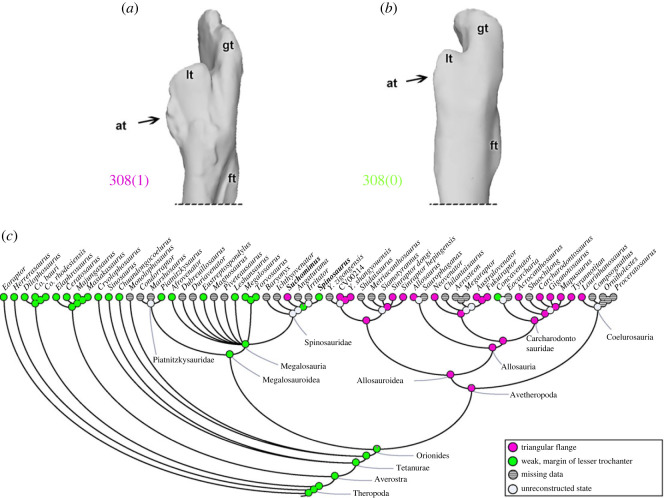
Evolutionary history of character 308 (femur, distinctly projecting accessory trochanter (derived from lesser trochanter)) and the ancestral state reconstruction. Illustration of the left femur in lateral view: (*a*) *Suchomimus* MNBH GAD500; (*b*) *Spinosaurus* FSAC-KK 11888. (*c*) Phylogenetic tree of Tetanurae showing the reconstruction of ancestral character state for each node. (*a*,*b*) Based on the three-dimensional digital model provided by Sereno *et al.* [16]. Not to scale. at, accessory trochanter; ft, fourth trochanter; gt, greater trochanter; lt, lesser trochanter.

A distinct anterodistal projection of the lesser trochanter, the accessory trochanter (character 308; e.g. [47]), may be a poorly developed structure that only forms a thickened distal margin of the lesser trochanter (308[0]) as in all non-avetheropods (except *Suchomimus*; figure 20*a*) analysed, including megalosauroids (e.g. *Spinosaurus*; figure 20*b*). Within avetheropods, the accessory trochanter represents a lateralized triangular projection (308[1]) except the carcharodontosaurid *Concavenator* which presents the plesiomorphic condition. Although *Suchomimus* has the apomorphic condition of a triangular projection, the MRCA of megalosauroids was plesiomorphic (pl = 99%), whereas the MRCA of Averostra had the derived state (pl = 94%) (figure 20*c*). PIFI2 is thought to have inserted here [47,50], so the more derived states suggest at least slight alterations in PIFI2 muscle actions [83,84].

On the anterodistal surface of the femur, on the medial side of the *M*. *femorotibialis externus* (FMTE; e.g. [47,73]) origin (character 309), the scar is small and rough (309[0]) in *Dilophosaurus* and early tetanurans including Megalosauroidea [6]. In other theropods, including ceratosaurs and avetheropods, this part of the origin of the FMTE is marked by a rough and pronounced oval depression, which extends distally (309[1]). Megalosauroids have the plesiomorphic condition [6], being the feature at the MRCA (pl = 96%), whereas derived Orionides such as allosaurs have the apomorphic condition (state 1 in the MRCA; pl = 93%). This is an interesting, persistent trait that probably has some implications for the biomechanics of the FMTE muscle; perhaps at least its size.

Distally, the medial epicondyle of the femur (character 310) may be rounded in shape (310[0]), as in most early theropods (except ceratosaurs; [98]) including megalosauroids. Yet this character varies widely, because in *Co*. *rhodesiensis*, ceratosaurs, early tetanurans and allosauroids (except *Saurophaganax*), the medial epicondyle is a bony crest (310[1]). This character's state is ambiguous for the MRCA of Orionides (pl = 52% for state 0), although the MRCA of megalosauroids had the plesiomorphic condition (pl = 95%).

**Figure 21.**
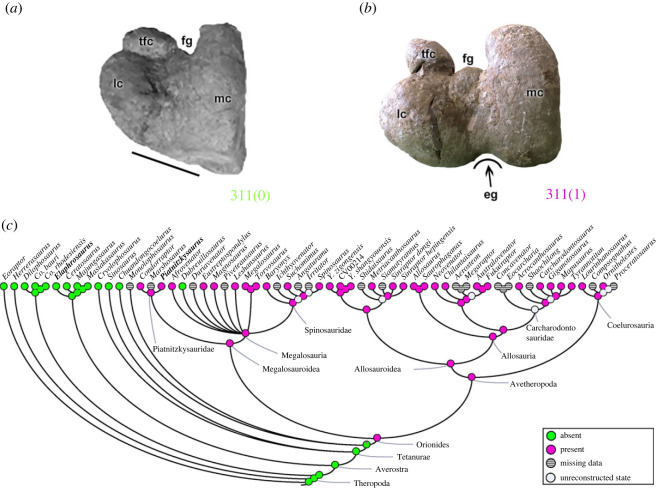
Evolutionary history of character 311 (femur, distal extensor groove) and the ancestral state reconstruction. Illustration of the left femur in distal view: (*a*) *Elaphrosaurus* MB R 4960; (*b*) *Piatnitzkysaurus* PVL 4073. (*c*) Phylogenetic tree of Tetanurae showing the reconstruction of ancestral character state for each node. (*b*) Modified from [89]. Not to scale. eg, extensor groove; fg, flexor groove; lc, lateral condyle; mc, medial condyle; tfc, tibiofibular crest.

The distal end of the femur, on the anterior surface dividing the medial and lateral condyles, may have a distal extensor groove (character 311), which is correlated with *triceps femoris* muscle paths [47]. This groove may be absent (311[0]), such as in non-Orionides theropods (e.g. *Elaphrosaurus*; figure 21*a*) and the megalosauroid *Dubreuillosaurus*. All other Orionides have the extensor groove (311[1]) (e.g. *Piatnitzkysaurus*; figure 21*b*). This character represents a rare change; the MRCA of Orionides had the distal extensor sulcus (pl = 97%) (figure 21*c*).

**Figure 22.**
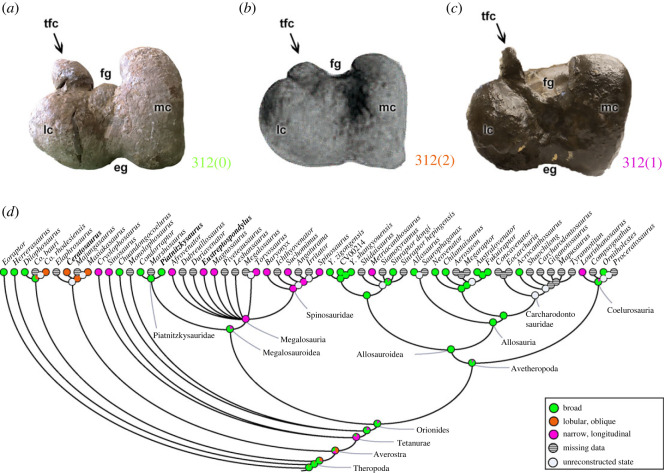
Evolutionary history of character 312 (femur, morphology and orientation of tibiofibularis crest) and the ancestral state reconstruction. Illustration of the left femur in distal view: (*a*) *Piatnitzkysaurus* PVL 4073; (*b*) *Ceratosaurus* SHN(JJS)-65/1; (*c*) *Eustreptospondylus* OUMNH J.13558/F02. (*d*) Phylogenetic tree of Tetanurae showing the reconstruction of ancestral character state for each node. (*b*) Modified from [100]. Not to scale. eg, extensor groove; fg, flexor groove; lc, lateral condyle; mc, medial condyle; tfc, tibiofibular crest.

The tibiofibular crest (or crista tibiofibularis; [23]) of the femur (character 312) may represent an enlarged structure (312[0]), as in several taxa including *Eoraptor*, *Herrerasaurus*, *Dilophosaurus*, piatnitzkysaurids (e.g. *Piatnitzkysaurus*; figure 22*a*) and avetheropods (except *Metriacanthosaurus* and *Lourinhanosaurus*). In early diverging tetanurans, megalosauroids (except piatnitzkysaurids) and aforementioned avetheropods, the tibiofibular crest is narrow and longitudinally oriented (312[1]) (e.g. *Eustreptospondylus*; figure 22*c*). In non-tetanurans such as *Co*. *rhodesiensis* and ceratosaurs (e.g. *Ceratosaurus*; figure 22*b*), this crest is a lobular ridge that is obliquely oriented (312[2]). Piatnitzkysaurids have a broad tibiofibular crest, whereas other megalosauroids have a narrow and longitudinal crest. Consequently, the MRCA of megalosauroids may have had a broad crest (pl = 78%), whereas the MRCA of megalosaurids + spinosaurids had a narrow, longitudinal tibiofibular crest (pl = 99%) (figure 22*d*).

**Figure 23.**
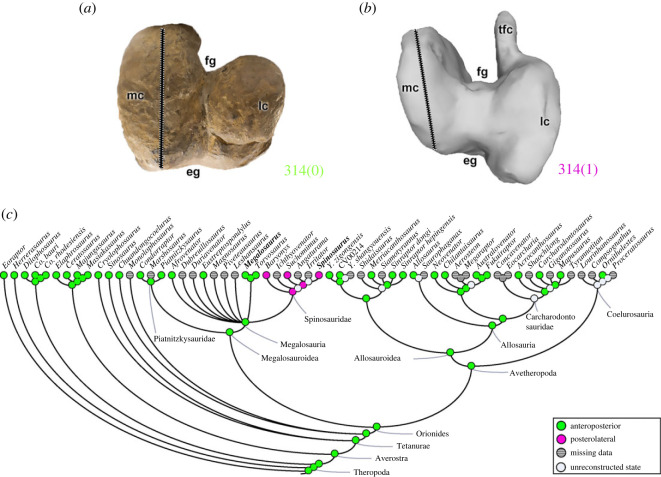
Evolutionary history of character 314 (femur, orientation of long axis of medial condyle in distal view) and the ancestral state reconstruction. Illustration of the right femur in distal view: (*a*) *Megalosaurus* OUMNH J.13561; (*b*) *Spinosaurus* FSAC-KK 11888. (*c*) Phylogenetic tree of Tetanurae showing the reconstruction of ancestral character state for each node. (*b*) Based on the three-dimensional digital model provided by Sereno *et al.* [16]. Not to scale. eg, extensor groove; fg, flexor groove; lc, lateral condyle; mc, medial condyle; tfc, tibiofibular crest.

A connection between the distal part of the medial femoral condyle and the tibiofibular crest, the infrapopliteal crest (character 313; e.g. [101]), may be absent (313[0]), as in all non-ceratosaur theropods, or present (313[1]) in ceratosaurs. The orientation of the main axis of the medial femoral condyle (character 314) can be arranged anteroposteriorly (314[0]), as observed conservatively in almost all theropod clades (e.g. *Megalosaurus*; figure 23*a*). The exception is spinosaurid taxa (e.g. *Spinosaurus*; figure 23*b*), in which the orientation of the medial condyle is in the posterolateral direction (314[1]), being the condition in the MRCA (pl = 98%) (figure 23*c*).

**Figure 24.**
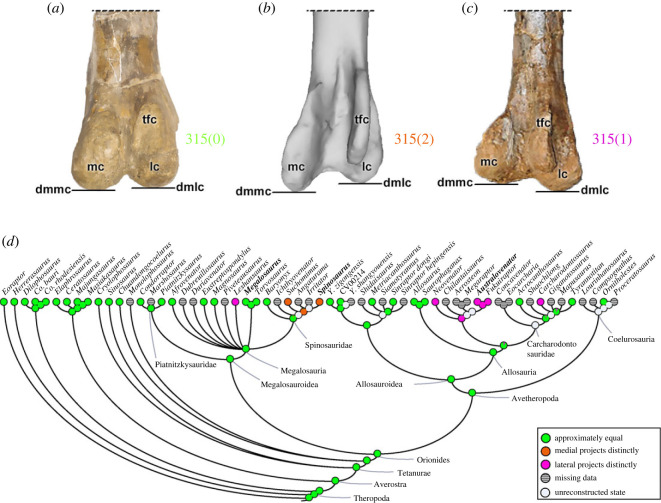
Evolutionary history of character 315 (femur, projection of lateral and medial distal condyles) and the ancestral state reconstruction. Illustration of the right femur in posterior view: (*a*) *Megalosaurus* OUMNH J.13561; (*b*) *Spinosaurus* FSAC-KK 11888; (*c*) *Australovenator* AODF604. (*d*) Phylogenetic tree of Tetanurae showing the reconstruction of ancestral character state for each node. (*b*) Based on the three-dimensional digital model provided by Sereno *et al.* [16] and (*c*) modified from [97]. Not to scale. dmlc, distal margin of lateral condyle; dmmc, distal margin of medial condyle; lc, lateral condyle; mc, medial condyle; tfc, tibiofibular crest.

The medial and lateral condyles of the femur may have their projection (character 315) approximately equal (315[0]), as seen in most theropods (e.g. *Megalosaurus*; figure 24*a*). A lateral condyle that projects beyond the medial condyle, with the distal surface of the medial condyle slightly flattened (315[1]), is a feature independently evolved in the megalosauroid *Leshansaurus*, neovenatorids (e.g. *Australovenator*; figure 24*c*) and *Carcharodontosaurus*. A medial condyle that projects distinctly further than the lateral (315[2]) exists only in *Suchomimus* and *Spinosaurus* (figure 24*b*). Even with some internal variations, the MRCA of megalosauroids (pl = 99%) and spinosaurids (pl = 96%) had condyles that projected equally (figure 24*d*).

The distal end of the femur (character 316) may present a centralized posterior depression, which is connected to the tibiofibular crest by a narrow groove (316[0]), as in non-avetheropod taxa. Within avetheropods (except *Y*. *zigongensis*), the depression separating the lateral and medial convexities is shallow and anteroposteriorly oriented (316[1]).

These changes of the morphology of the distal femur (characters 310–316) pertain to the femorotibial and femorofibular joints (i.e. knee), and surely would influence the kinematics of those articulations (e.g. [92]). Some may also relate to ligaments connecting the three long bones involved in the knee. More investigation of knee form and function in theropods (e.g. [102]) is needed to understand such traits.

#### 3.2.7. Zeugopodium (tibia)

Eight morphological characters (11.76%) relate to the tibia. The proximal tibia in theropods and crocodylians is where several muscles of the thigh such as IT1–3, AMB, FMT, FTI3 and FTE or their homologues are/were attached; as well as the origin of EDL and *M*. *gastrocnemius pars medialis* (GM) (e.g. [50,69,70,72,73,90]).

The lateral malleolus of the distal tibia (character 317) can be positioned posterior to the astragalus (317[0]), as in early theropods. The overall feature of averostran theropods (except *Cryolophosaurus*) is a lateral malleolus that overlaps the calcaneum (317[1]). The shape of the lateral malleolus's edge (character 318) is conservative, being smoothly curved (318[0]) in all non-coelophysoid theropods, whereas coelophysoids present a tubular notch (318[1]). Perhaps characters 317 and 318 indicate reduced mobility of the tibiotarsal joints with derived states.

**Figure 25.**
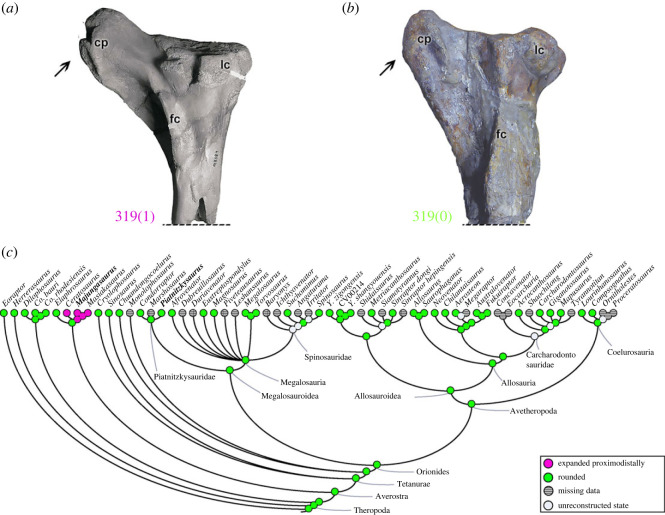
Evolutionary history of character 319 (tibia, morphology of distal cnemial process) and the ancestral state reconstruction. Illustration of the left tibia in lateral view: (*a*) *Majungasaurus* FMNH PR 2424; (*b*) *Piatnitzkysaurus* MACN-Pv-CH 895. (*c*) Phylogenetic tree of Tetanurae showing the reconstruction of ancestral character state for each node. (*a*) Modified from [103]. Not to scale. cp, cnemial process; fc, fibular crest; lc, lateral condyle.

The cnemial process or cnemial crest (e.g. [95]) on the proximal tibia of theropods has its distalmost morphology (character 319) rounded (319[0]) in all non-ceratosaurid theropods analysed (e.g. *Piatnitzkysaurus*; figure 25*b*). The feature in ceratosaurids (e.g. *Majungasaurus*; figure 25*a*) is a proximodistally expanded cnemial crest (319[1]). This derived expansion suggests an expanded set of tendinous insertions of the *triceps femoris* knee extensor muscles (IT1-3, AMB, *Mm*. *femorotibiales*).

The lateral condyle/cotyle of the tibia (or fibular condyle; [95]) (character 320) may be large (320[0]) as in all non-avetheropods (except *Suchomimus*), or small and lobular (320[1]), as in avetheropods (except *Sinraptor*) and *Suchomimus*. The derived condition present in *Suchomimus* represents a secondary acquisition, because the MRCA of megalosauroids had a large lateral condyle of the tibia (pl = 99%). An anterolateral process of the lateral tibial condyle/cotyle (character 321) may be absent or represent a horizontal projection (321[0]), as in all non-neovenatorid theropods. A prominent and ventrally curved process (321[1]) is a synapomorphy of neovenatorids. While suggestive of changes in knee joint function, it is difficult to even speculate on what those changes might be, as the function of the tibiofibular side of the knee joint in archosaurs is even more poorly understood than that of the femoral side.

In the distal tibia, the anteromedial buttress for the astragalus (supraastragalar buttress) (character 322) is absent (322[0]) in *Herrerasaurus*. A ventrally positioned anteromedial buttress (322[1]) exists in coelophysids. In most theropods analysed including ceratosaurs, early tetanurans, megalosauroids (except *Suchomimus*) and non-carcharodontosaurian avetheropods, the anteromedial buttress is a marked oblique step-like ridge (322[2]). In carcharodontosaurids and *Neovenator*, the anteromedial buttress is a reduced oblique ridge (322[3]). Meanwhile, neovenatorids (except *Neovenator*), and the spinosaurid *Suchomimus* have the bluntly rounded vertical ridge on the medial side of the anteromedial buttress (322[4]). Despite the derived state in *Suchomimus*, the MRCA of megalosauroids had a marked oblique step-like ridge related to the anteromedial tibial buttress (pl = 99%). Like characters 317 and 318, this character may signal reduction of mobility.

**Figure 26.**
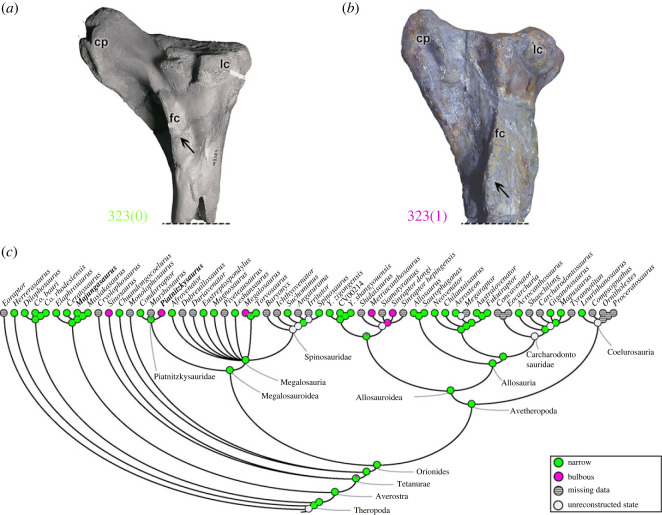
Evolutionary history of character 323 (tibia, morphology of fibular crest) and the ancestral state reconstruction. Illustration of the left tibia in lateral view: (*a*) *Majungasaurus* FMNH PR 2424; (*b*) *Piatnitzkysaurus* MACN-Pv-CH 895. (*c*) Phylogenetic tree of Tetanurae showing the reconstruction of ancestral character state for each node. (*a*) Modified from [103]. Not to scale. cp, cnemial process; fc, fibular crest; lc, lateral condyle.

On the proximal tibia, the morphology of the fibular crest, or crista fibularis (character 323), is narrow (323[0]) in most of the analysed theropods (e.g. *Majungasaurus*; figure 26*a*). Exceptions are when the crista fibularis becomes a bulbous structure (323[1]), as in *Sinosaurus*, *Piatnitzkysaurus* (figure 26*b*), *Megalosaurus* and some metriacanthosaurids. Even with the derived state of a bulbous crest in *Piatnitzkysaurus* and *Megalosaurus*, the MRCA of megalosauroids had a narrow structure (pl = 99%) (figure 26*c*). However, one study concluded that *Suchomimus* has a large and bulbous crest ([104] *contra* [6]).

**Figure 27.**
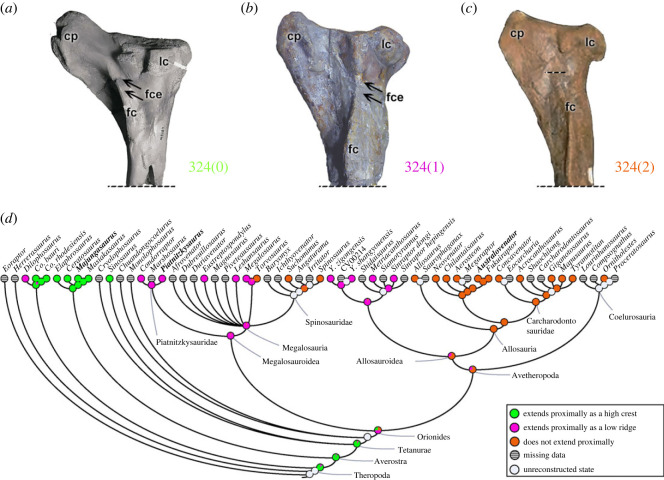
Evolutionary history of character 324 (tibia, development of fibular crest) and the ancestral state reconstruction. Illustration of the left tibia in lateral view: (*a*) *Majungasaurus* FMNH PR 2424; (*b*) *Piatnitzkysaurus* MACN-Pv-CH 895; (*c*) *Australovenator* AODF604. (*d*) Phylogenetic tree of Tetanurae showing the reconstruction of ancestral character state for each node. (*a*) Modified from [103] and (*c*) modified from [97]. Not to scale. cp, cnemial process; fc, fibular crest; fce, fibular crest extension; lc, lateral condyle.

The fibular crest development (character 324) in some early theropods such as coelophysoids and ceratosaurs (e.g. *Majungasaurus*; figure 27*a*) is proximally high, extending to the proximal end of the tibia (324[0]). In megalosauroids (except *Torvosaurus* and spinosaurids) and metriacanthosaurids, the fibular crest extends to the proximal end of tibia as a low ridge (324[1]) (e.g. *Piatnitzkysaurus*; figure 27*b*). Non-metriacanthosaurid averostrans and *Torvosaurus* + spinosaurids have a fibular crest that does not extend to the proximal end of the tibia (324[2]) (e.g. *Australovenator*; figure 27*c*). Because most of the megalosauroids have a low ridge fibular crest, the MRCA had this state (pl = 94%) (figure 27*d*). The crista tibiofibularis is considered to indicate strengthening of the attachment between the two zeugopodial bones, enhanced action of the ILFB and perhaps more (reviewed in [49]).

#### 3.2.8. Zeugopodium (fibula)

At least four morphological characters (5.88%) relate to the fibula. Some muscles such as *M*. *fibularis longus* (FL), *M*. *fibularis brevis* (FB) and *M*. *extensor hallucis longus* (EHL) are shared among crocodylians, Aves and non-avian theropods and originate(d) from the fibula; whereas the ILFB inserts here [49,50,69,70].

The depth of the fibular fossa on the medial fibula (character 325) may be a groove (325[0]) as in coelophysoids and *Sinosaurus*; or a shallow fossa (325[1]) that is present only in spinosaurids and megalosaurids. Therefore, a deep fossa (325[2]) is acquired independently in ceratosaurs, *Chuandongocoelurus*, piatnitzkysaurids and averostran theropods. Although a shallow fossa is a widespread feature among megalosauroids (most parsimonious for the MRCA of Megalosauria), the most parsimonious condition for the MRCA of megalosauroids is the presence of a deep fossa. The fibular fossa on the fibula can be positioned (character 326) posteriorly (326[0]), as in coelophysoids, or centrally (326[1]), as seen widely in neotheropods. This fossa might be a more concentrated origin of part of the digital flexors, or part of the ‘popliteus’/interosseous cruris/pronator profundus (see [49,90]).

**Figure 28.**
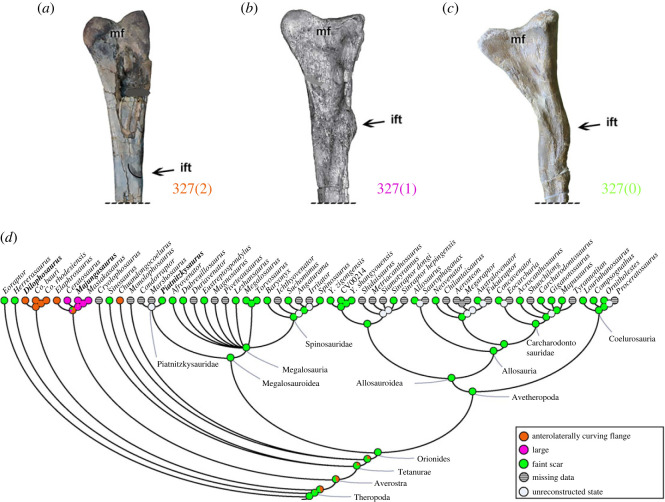
Evolutionary history of character 327 (fibula, size of iliofibularis tubercle) and the ancestral state reconstruction. Illustration of the left fibula in medial view: (*a*) *Dilophosaurus* TMM 43646-1; (*b*) *Majungasaurus* FMNH PR 2424; (*c*) *Piatnitzkysaurus* PVL 4073. (*d*) Phylogenetic tree of Tetanurae showing the reconstruction of ancestral character state for each node. (*a*) Modified from [82] and (*b*) modified from [103]. Not to scale. ift, iliofibularis tubercle; mf, medial fossa.

The shape of the iliofibularis tubercle (character 327) that is widespread within tetanuran theropods is a faint scar (327[0]) (e.g. *Piatnitzkysaurus*; figure 28*c*). In ceratosaurs (except *Elaphrosaurus*), the tubercle is a large structure (327[1]) (e.g. *Majungasaurus*; figure 28*b*); and the morphology in coelophysoids (e.g. *Dilophosaurus*; figure 28*a*), *Elaphrosaurus* and *Chuandongocoelurus* is an anterolaterally curved flange (327[2]).

The size of the proximal fibula relative to the width of the proximal tibia (character 328) is less than 75% (328[0]) only in *Eoraptor*; all theropods analysed here have this proportion greater than or equal to 75% (328[1]). This difference may indicate the enlarged tibia (and knee extensor insertions) in early theropods; along with part of the protracted trend of reduction of the fibula along the lineage to Aves.

### 3.3. Morphological disparity

To illustrate the morphological dissimilarity of the theropod locomotor apparatus between taxonomic groups, on the basis of morphological variation, we sectioned our results based on different locomotor elements, displayed in a two-dimensional morphospace sorting the Euclidean distances between taxa.

#### 3.3.1. Pelvic girdle and hindlimb stylopodium and zeugopodium

**Figure 29.**
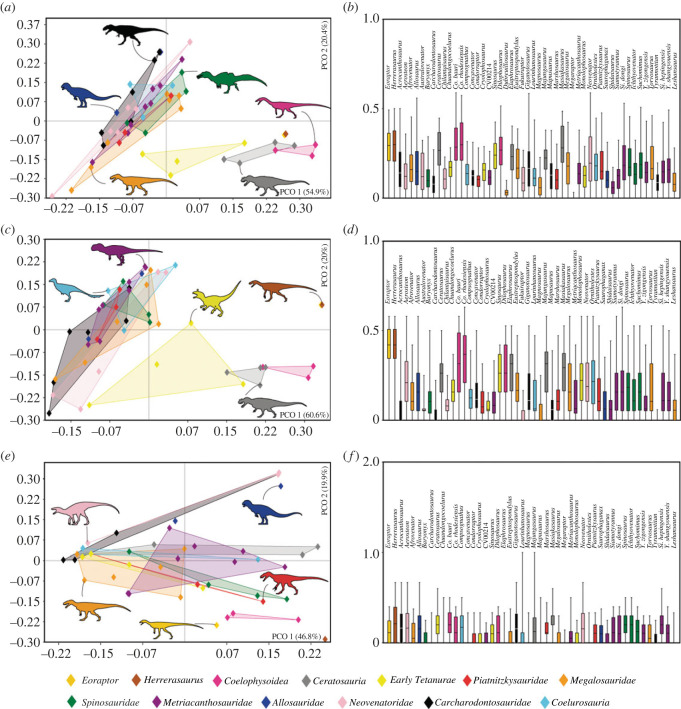
Two-dimensional morphospace and box plot diagrams based on Euclidean taxon–taxon distance related to morphological characters of the theropod locomotor system. (*a*) PCO1 versus PCO2 biplot (75.3% of variance) and (*b*) box plot diagram of pelvic girdle and hindlimb's stylopodium and zeugopodium (characters 261–328). (*c*) PCO1 versus PCO2 biplot (80.6% of variance) and (*d*) box plot diagram of ilium (characters 262–280). (*e*) PCO1 versus PCO2 biplot (66.7% of variance) and (*f*) box plot diagram of pubis (characters 282–291). Silhouettes were downloaded from phylopic.org; see Acknowledgements.

In the morphological disparity analysis considering all the characters related to the pelvic girdle and stylopodium/zeugopodium, the morphospace with the highest variance accumulates 75.3% of the data dissimilarity. The PCO1 versus PCO2 (figure 29*a*) reveals the overlap of several lineages: *Eoraptor*, *Herrerasaurus*, coelophysoids, ceratosaurs and most of the early diverging tetanurans occupy positive PCO1 and negative PCO2 scores. As expected, non-Orionides occupy different positions in morphospace when compared with Orionides, indicating more dissimilarity (also confirmed in box plots, e.g. *Coelophysis*; figure 29*b*). A biplot (figure 29*a*) indicates that the Orionides clade is distributed in a similar way in the morphospace, since there is a high degree of overlap of several groups including megalosauroids, allosauroids and coelurosaurs; however, with the main groups distributed along five main axes with a high degree of overlap between piatnitzkysaurids, megalosaurids and a moderate overlap with spinosaurids. Considering the PCO2 axis, the clades that occupy the largest area in morphospace are the neovenatorids, metriacanthosaurids, megalosaurids and carcharodontosaurids, with a high to moderate degree of overlap between clades. Megalosauroidea are distributed in a similar way as other Orionides clades in the biplot; however, megalosaurids, based on the convex hulls, have a larger occupation of the morphospace (influenced most by the negatively scored taxa, *Dubreuillosaurus* and *Magnosaurus*).

#### 3.3.2. Ilium

In the biplot focusing on the morphological characters of the ilium (PCO1 versus PCO2 = 80.6% of variance; figure 29*c*), the taxon distribution is nearly similar to the complete dataset analysis (i.e. pelvis + stylopodium/zeugopodium; figure 29*a*). At the most positive scores of PCO1, *Eoraptor* and *Herrerasaurus* completely overlap, and the morphospace is gradually occupied along the PCO1 (to negative scores) by coelophysoids, ceratosaurs and early diverging tetanurans (which occupy a large morphospace area), indicating more dissimilarity to Orionides (figure 29*c*,*d*). More negative scores of PCO1 have a similar distribution of the major Orionides clades distributed along a main axis with the overlap of several clades. The early diverging tetanurans, carcharodontosaurids, megalosaurids and neovenatorids cover a great area of the morphospace. Considering the PCO2 axis, megalosauroids expanded their morphospace distribution compared with the complete dataset analysis. The Orionides clade have a broad distribution along the PCO2 axis; however, this clade is almost restricted to negative scores along PCO1. Our analysis considering the morphological characters of the ilium shows that they have a strong influence on the disparity metrics quantified by the complete dataset analysis (pelvis + stylopodium/zeugopodium), because they contain many studied characters (27.94%) and both biplots present a similar pattern.

#### 3.3.3. Pubis

When we consider only the pubis, the non-theropod *Eoraptor* and coelophysoids retain extreme negative scores for PCO2 and positive scores for PCO1 in the morphospace (PCO1 versus PCO2 = 66.7% of variance; figure 29*e*). On the other hand, averostran taxa are differently distributed in the morphospace, mainly due to the influence of the PCO2 axis that segregates these taxa from non-averostrans, and distributes them approximately across five main axes according to the clade-based delimitations of the convex hulls (figure 29*e*). The most positive scores along PCO1 reached by ceratosaurs are influenced by *Masiakasaurus* and *Ceratosaurus* distribution; but the clade has a nearly homogeneous distribution along the PCO2 axis. Spinosaurids, piatnitzkysaurids and early diverging tetanurans have similar patterns for their morphospace distributions, with spinosaurids being more positively scored along both axes. Allosauria, including carcharodontosaurids, neovenatorids and allosaurids, reaches the most positive scores in the PCO2 in similar pattern, but distributed differently when compared with ceratosaurs and early tetanurans. The clades occupying larger areas in the morphospace are megalosaurids, which are restricted to negative scores of PCO1; and metriacanthosaurids, which overlap with several other clades such as coelurosaurs, spinosaurids and ceratosaurs.

#### 3.3.4. Ischium

**Figure 30.**
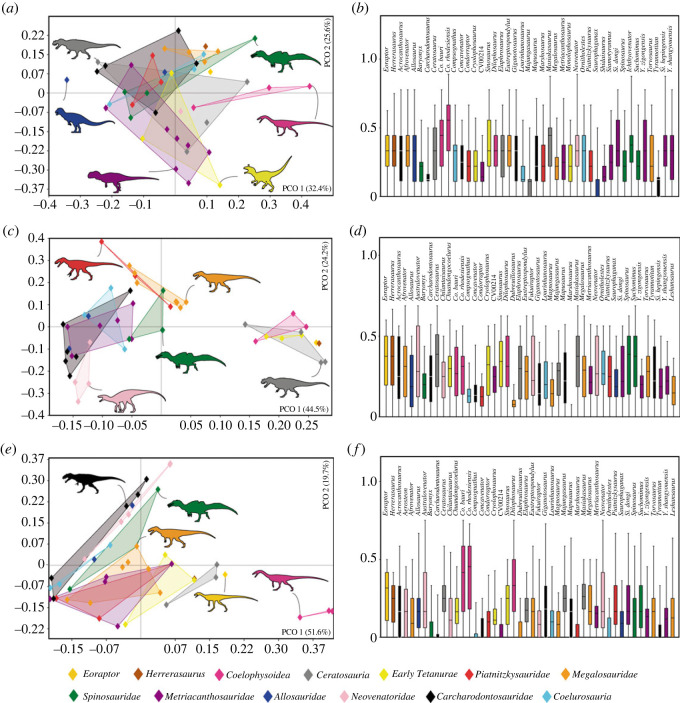
Two-dimensional morphospace and box plot diagrams based on Euclidean taxon–taxon distance related to morphological characters of the theropod locomotor system. (*a*) PCO1 versus PCO2 biplot (58% of variance) and (*b*) box plot diagram of ischium (characters 292–300). (*c*) PCO1 versus PCO2 biplot (68.7% of variance) and (*d*) box plot diagram of stylopodium (characters 301–316). (*e*) PCO1 versus PCO2 biplot (71.3% of variance) and (*f*) box plot diagram of zeugopodium (tibia and fibula) (characters 317–328). Silhouettes were downloaded from phylopic.org; see Acknowledgements.

In our analysis of the ischium, for the morphospace with greatest morphological variance (PCO1 versus PCO2 = 58%; figure 30*a*), the coelophysoid clade retains the most positive scores for the PCO1; influenced by *Coelophysis* (confirmed in box plot; figure 30*b*). The remaining taxa are distributed along five main axes, with a high degree of overlap. Ceratosaurs, early tetanurans, metriacanthosaurids, carcharodontosaurids and spinosaurids occupy large areas of the morphospace; with ceratosaurs, metriacanthosaurids and early tetanurans retaining negative PCO2 scores, and remaining clades having more positive scores (figure 30*a*). Compared with our previous analyses above, there is a large relative increase of the morphospace area occupied by several groups, for example, metriacanthosaurids, early tetanurans, ceratosaurs, carcharodontosaurids, piatnitzkysaurids and spinosaurids.

#### 3.3.5. Stylopodium

By analysing only the influence of the femur on the morphospace (PCO1 versus PCO2 = 68.7% of variance; figure 30*c*), we find that the non-Orionides taxa *Herrerasaurus*, coelophysoids, ceratosaurs and early tetanurans occupy positive scores for PCO1, close together. Meanwhile, Orionides has mainly negative scores for PCO1, although some megalosauroids (e.g. *Torvosaurus*, *Piatnitzkysaurus*, *Spinosaurus*) have slightly positive scores along this axis. There is great overlap between avetheropods for the most negative PCO1 scores; by coelurosaurs, allosaurs, metriacanthosaurids and carcharodontosaurids. In this analysis, the distribution in morphospace is consistent with the phylogeny of theropods, with the delimited clades occupying different areas: non-Orionides (*Herrerasaurus*, coelophysoids, ceratosaurs and early tetanurans) retaining positive scores for PCO1 and close to the average or slightly negative for PCO2; megalosauroids (piatnitzkysaurids, megalosaurids and spinosaurids) retaining near average to slightly negative PCO1 scores and from positive to near average PCO2 scores; and avetheropods retaining negative PCO1 scores and ranging from positive to negative PCO2 scores, with the neovenatorid theropods being the most negatively positioned along PCO2 (figure 30*c*,*d*).

#### 3.3.6. Zeugopodium

In our analysis of the zeugopodium, for the morphospace with greatest morphological variance (PCO1 versus PCO2 = 71.3%; figure 30*e*), almost all non-Orionides taxa retain positive PCO1 scores, with coelophysoids isolated to extreme positive values (and consequently larger box plots; figure 30*f*), followed by ceratosaurs. Regarding tetanurans, five main distribution axes are evident based on the divisions of clades: piatnitzkysaurids, the majority of megalosaurids and metriacanthosaurids converge in the morphospace, as well as carcharodontosaurids, coelurosaurs and neovenatorids that are distributed close together; however, neovenatorids reach extreme PCO2 scores that seem influenced by *Australovenator* and *Neovenator* (figure 30*e*,*f*). Both megalosaurids and metriacanthosaurids occupy a large area in the morphospace. Spinosaurids occupy a distinct and large morphospace area, influenced by *Suchomimus*.

### 3.4. Alternative phylogenies and the potential validity of the Carnosauria

Some recent phylogenies have recovered Carnosauria (i.e. Megalosauroidea + Allosauroidea; *sensu* [57]) as a clade (e.g. [20,57,105]), which would have important implications for the early evolution of tetanurans. Carnosauria is defined as a clade that includes all theropods that are more closely related to *Allosaurus* and to *Megalosaurus* than to Neornithes [57]. Previously, most analyses recovered three distinct clades: Megalosauroidea, Allosauroidea and Coelurosauria, with the latter two traditionally recovered a sister group (Avetheropoda clade); and Megalosauroidea being rootward to Avetheropoda (e.g. [6,8,13,23,106]; this work). In the analysis of Rauhut & Pol [57], Spinosauridae was the first group of tetanurans to diverge, followed by Megalosauridae and Piatnitzkysauridae (the latter being allocated in Allosauroidea), thus with Megalosauridae as the sister group of Allosauroidea. Barker *et al*. [20] obtained similar results, but Allosauroidea (+ Piatnitzkysauridae) species generally formed only a polytomy. However, in contrast with Rauhut & Pol [57], the clade composed of Megalosauridae and Spinosauridae (i.e. Megalosauria) was recovered. Schade *et al*. [105] also found a monophyletic Carnosauria, but in contrast to the previous hypotheses, the taxa classically considered as Megalosauridae formed a grade outside of Spinosauridae, and Piatnitzkysauridae was placed as an Allosauroidea clade. These studies' main implications (in terms of evolution of pelvic and appendage characters) contrasting with our results are as follows.

(1) Character 263; ilium, vertical ridge on the lateral surface of blade dorsal to acetabulum. In our results, the presence of a low swollen ridge (263[1]) converges between some megalosauroids (including Piatnitzkysauridae) and Allosauridae. Considering Carnosauria, the presence of a low swollen ridge could have arisen in the MRCA of Allosauroidea (and homoplastically in the megalosauroids *Afrovenator*, *Megalosaurus* and *Suchomimus*), and later having been lost (263[0]) or expanded (263[2]) in late allosauroids.
(2) Character 269; ilium, shape of acetabular margin of pubic peduncle. Although it is a relatively homoplastic character, in our results an acetabular margin convex or flat (269[0]) was a feature of Piatnitzkysauridae that would potentially be present in the MRCA of megalosauroids. In the Carnosauria hypothesis, in which Piatnitzkysauridae is a member of Allosauroidea, this condition would have been independently acquired in *Eoraptor*, *Herrerasaurus*, ceratosaurs, *Spinosaurus* and predominant in Allosauroidea.
(3) Character 281; puboischiadic plate, morphology, and foramina/notches. Although with some variations (mainly in *Yangchuanosaurus*), in our results, the presence of an open midline without fenestrae and 1–2 notches (281[2]) is predominant and the condition for the MRCA of Avetheropoda; also present homoplastically in *Afrovenator*. In the Carnosauria hypothesis, this condition (i.e. 281[2]) was present in the MRCA of Tetanurae, considering what is observed in Coelurosauria, and later in Megalosauria reverted to the condition of being fully closed along midline with three fenestrae (281[0]) (except *Afrovenator* and *Leshansaurus*); also independently in Piatnitzkysauridae.
(4) Character 292; ischium, length relative to pubis length. Contrary to our results, in the Carnosauria hypothesis, the acquisition of the ischium length relative to the pubis greater than 80% (292[2]) would be characteristic of the clade formed by Metriacanthosauridae + Carcharodontosauria (except *Neovenator*) and independently acquired in *Sinosaurus*, *Torvosaurus* and *Spinosaurus*; rather than an acquisition of the MRCA from Allosauroidea, because Piatnitzkysauridae presents the plesiomorphic condition of this character (i.e. 75–80%; (292[0])).
(5) Character 297; ischium, morphology of symphysis. Although this character is homoplastic, we hypothesize that the presence of unexpanded symphysis (297[0]) in Piatnitzkysauridae (except *Marshosaurus*) was present in the MRCA of Megalosauroidea, modified in Megalosauria (to an expanded apron (297[1])) and later reversed in Spinosauridae (except *Ichthyovenator*). In the Carnosauria hypothesis, the plesiomorphic condition observed in Piatnitzkysauridae would potentially be shared between this group and Metriacanthosauridae (both at the base of Allosauroidea outside of Allosauria).
(6) Character 303; femur, groove on proximal surface of head-oriented oblique to long axis of head (articular groove or fovea capitis). In our results, a clear step in the acquisition of the articular groove or fovea capitis (303[1]) is noted in the MRCA of neotheropods, having been reversed (303[0]) in the MRCA of avetheropods. In the Carnosauria hypothesis, this scenario would be more complicated, and the loss of this structure would converge between Coelurosauria and non-Piatnitzkysauridae allosauroids.
(7) Character 308; femur, distinctly projecting accessory trochanter (derived from lesser trochanter). Based on our results, the presence of an accessory trochanter such as a triangular flange (308[1]) is a shared condition among avetheropods (reversed in *Concavenator* and acquired independently in *Suchomimus*). In the Carnosauria hypothesis, Coelurosauria and non-Piatnitzkysauridae allosauroids (and *Suchomimus*) would converge in the acquisition of the triangular flange; whereas Piatnitzkysauridae (as early allosauroids) would diverge from other allosauroids due to their weak and slightly thickened margin of the lesser trochanter (308[0]).
(8) Character 309; femur, *M*. *femorotibialis externus* origin medially on anterodistal surface. In our results, megalosauroids converge with *Dilophosaurus*, *Sinosaurus* and *Chuandongocoelurus* in the presence of a faint, small rugose patch (309[0]), whereas Allosauroidea (including the MRCA) have a pronounced rugose depression that extends to the distal femur (309[1]). Considering the Carnosauria hypothesis, the placement of Piatnitzkysauridae at the base of Allosauroidea would suggest this represented the plesiomorphic allosauroid condition for this character, later modified in non-Piatnitzkysauridae allosauroids.
(9) Character 310; femur, development of medial epicondyle. Our results find the presence of a ridge (310[1]) as convergent between *Coelophysis*, ceratosaurs, early tetanurans and allosauroids (except *Saurophaganax*). In the Carnosauria hypothesis, not all allosauroids would have this condition, because Piatnitzkysauridae has a rounded medial epicondyle (310[0]).
(10) Character 316; femur, morphology of distal end. Our results indicate that the acquisition of an anteroposteriorly oriented shallow trough separating the medial and lateral convexities on the distal end of the femur (316[1]) evolved in the MRCA of avetheropods (except *Yangchuanosaurus*). In the Carnosauria hypothesis, this scenario becomes more complex: this feature (i.e. 316[1]) would have arisen independently in Coelurosauria and non-Piatnitzkysauridae allosauroids.
(11) Character 325; fibula, depth of fibular fossa on medial aspect. Although there is some homoplasy in our results, a deep fossa (325[2]) was present in Tetanurae (except Megalosauria, which presents a shallow fossa (325[1])). Even in the Carnosauria hypothesis, the interpretation is similar, because Piatnitzkysauridae shares the same condition (i.e. 325[2]) with Allosauroidea and Coelurosauria.

### 3.5. Summary of results

First, our phylogeny recovers piatnitzkysaurids as the first clade to diverge among megalosauroids, then a succession of taxa represented by a polytomy among megalosaurids, but with *Megalosaurus* and *Torvosaurus* being closely related, and then spinosaurids having *Baryonyx* as the first branch of divergence and *Suchomimus* as the outgroup of Spinosaurinae.

Second, we reveal key morphological transitions within/at Megalosauroidea. During the evolution of megalosauroids, there was (i) the mosaic emergence of a low swollen ridge on the ilium (in piatnitzkysaurids, *Afrovenator*, *Megalosaurus* and *Suchomimus*); (ii) enlargement of the posterior portion of the brevis fossa (in *Marshosaurus*, *Eustreptospondylus* and spinosaurids except *Ichthyovenator*); (iii) the anterior wall of the brevis fossa became taller along its whole length in *Suchomimus* and *Spinosaurus*; (iv) emergence of a prominent posterodorsal process on the ilium in some megalosaurids; (v) changes in the orientation of the pubis shaft, becoming ventrally concave in *Marshosaurus* and dorsally concave in *Spinosaurus*; (vi) the ischial shafts became ventrally curved in some megalosaurids; (vii) origin of a femoral head that is anteromedially oriented and medially angulated; (viii) a narrow and longitudinal tibiofibularis crest in non-piatnitzkysaurid megalosauroids; (ix) appearance of a posterolaterally oriented medial condyle of the femur in spinosaurids; and (x) medial and lateral condyles that project distally in *Suchomimus* and *Spinosaurus*. The posterior width of brevis fossa and the morphology of ischial symphysis seems to be the most homoplastic features in megalosauroids. These and other traits have some functional relevance (as well as some unclear relevance) detailed above, and further considered below.

Third, we characterize how pelvic and hindlimb characters occupy different (or similar) regions of morphospace in Theropoda. The greatest dissimilarity in the ilium was in megalosaurids based on the large morphospace area—a high degree of homoplasy is suggested for this structure in Orionides. For the pubis, the greatest morphological variation occurs in piatnitzkysaurids and spinosaurids, and there is a distinction among coelophysoids, carcharodontosaurs and others theropods—the overlap between non-carcharodontosaur tetanurans suggests a moderate amount of pubic homoplasy. The largest ischial morphospace area is occupied by spinosaurids and ceratosaurs—we find a weak phylogenetic signal, suggesting abundant homoplasy. We uncover a clear distinction in the femoral morphospace distribution pattern regarding megalosauroids and other theropods, such as avetheropods and non-tetanurans (suggesting a strong phylogenetic signal). Finally, piatnitzkysaurids show the greatest dissimilarity of zeugopodial characters: a distinction in the morphospace is evident for carcharodontosaurs, ceratosaurs and coelophysoids, whereas overlaps occur mainly among megalosaurids, piatnitzkysaurids and neovenatorids, suggesting some homoplasy.

## 4. Conclusion

### 4.1. Phylogenetic inference

**Figure 31.**
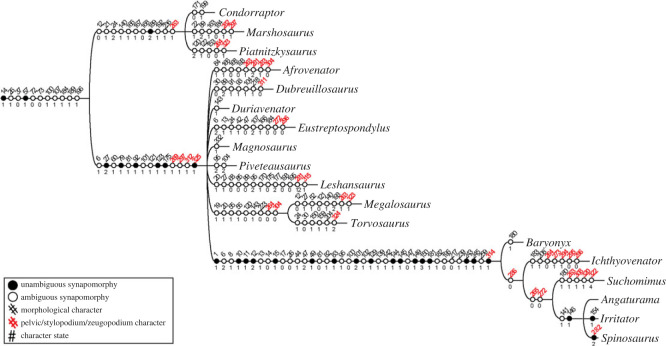
Mapped synapomorphies (unambiguous changes) for Megalosauroidea based on results retrieved from our phylogenetic analysis.

Our phylogenetic analysis conducted after inclusion of extra spinosaurid specimens, and reinterpretation of a few characters related to the locomotor system [6], recovered a monophyletic Megalosauroidea clade (figure 1) based on at least 11 synapomorphies (ambiguous and unambiguous) related to cranial and axial skeleton structures (figure 31). Similar to previous analyses (e.g. [6,8,19,53,54]), our phylogenetic inference includes taxa from the clades Piatnitzkysauridae, Megalosauridae and Spinosauridae in Megalosauroidea (however, see below).

Within Megalosauroidea, the first branch of divergence is the medium-sized species of the Piatnitzkysauridae clade (*Condorraptor*, *Marshosaurus* and *Piatnitzkysaurus*) (figure 1), which also have 11 synapomorphic features related to cranial structures and the axial and appendicular skeleton (figure 31), in addition to a synapomorphy of the locomotor system, which is the presence of a low swollen ridge on the lateral surface of the ilium (263[1]; figures 2 and 31). Our analysis recovers Piatnitzkysauridae as a polytomy for the three species of the clade, differing from previous analyses that usually recovered the North American Jurassic taxon *Marshosaurus* as an early diverging species, followed by a clade formed by the South American Jurassic forms *Condorraptor* + *Piatnitzkysaurus* (e.g. [6,57]). However, in other approaches (e.g. [8,23]), the Middle Chinese Jurassic taxon *Xuanhanosaurus* is recovered at the base of Piatnitzkysauridae. Instead, Carrano *et al*. [6] recovered *Xuanhanosaurus* within the avetheropod clade Metriacathosauridae, and considered it as a ‘wildcard’, so we did not include this taxon in our search. Even though our analysis is inconclusive about the internal evolutionary relationships of Piatnitzkysauridae, our inference supports that this clade represents the early Megalosauroidea clade, which in turn represents the first group of Tetanurae to diversify.

Our analysis recovered Megalosauria as a sister clade of Piatnitzkysauridae. Megalosauria is composed of the species traditionally placed in Megalosauridae and Spinosauridae. Megalosauria is supported by at least 14 synapomorphies (figure 31), four of which (three ambiguous and one unambiguous) related to the locomotor system: (i) transversely concave shape of acetabular margin of the ilium (269[1]; figure 8); (ii) morphology of the ischial symphysis expanded as apron (297[1]); (iii) narrow and longitudinal tibiofibularis crest of the femur (312[1]; figure 22); and the unambiguous (iv) shallow fossa on the medial position of the fibula (325[1]).

**Figure 32.**
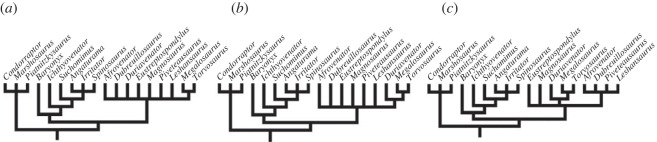
Majority rules consensus tree of Megalosauroidea considering the ‘required frequency of clades': (*a*) 85%; (*b*) 80% and (*c*) 60%.

Our search failed to retrieve Megalosauridae as a clade as previously defined [6]; instead, in our consensus topology the Jurassic species traditionally allocated in Megalosauridae represent a grade, with successive taxa representing outgroups to Spinosauridae (figure 1). Megalosauridae represented by a polytomy is not new in the literature (e.g. [30,54,107]; but see [6]); however, the group is based on previous diagnoses, based on cranial, axial and appendicular skeletal synapomorphies [6,7]. Nevertheless, when we adopt the majority rules consensus tree, considering 85% of the ‘required frequency of clades', we recover Megalosauridae as a clade (figure 32*a*); considering 80% of frequency, the Megalosaurinae clade is also recovered with the presence of *Duriavenator* at the base (figure 32*b*); and in the last approach, considering 60% of frequency, we recovered both monophyletic Megalosauridae, as well as Megalosaurinae and Afrovenatorinae (figure 32*c*)—similar to the results of Carrano *et al*. [6]. One of the synapomorphies of Megalosauridae hypothesized by Carrano *et al*. [6], related to the locomotor system, is the presence of a shallow groove on the posterior surface of the femur that demarcates the presence of the oblique ligament (304[0]). However, in our results (strict consensus tree) this condition seems to represent an independent acquisition in *Afrovenator* and in the clade composed by *Megalosaurus* + *Torvosaurus* (figure 31). Finally, although we failed to recover Megalosauridae, a clade composed by *Megalosaurus* + *Torvosaurus* is recovered (figure 1), somewhat equivalent with the clade Megalosaurinae (*sensu* [6]), however, without *Dubreuillosaurus*. This clade is supported by nine synapomorphies, two of which are related to the locomotor apparatus: 304[0] above, in addition to the presence of a brevis fossa whose posterior width is subequal to the anterior 264[0] (figures 3 and 31). A close evolutionary relation between *Megalosaurus* and *Torvosaurus* was corroborated by several approaches (e.g. [6,23,106]). If the polytomy represented by Megalosauridae recovered here and in previous studies (e.g. [54]) represents a soft or a hard polytomy, future efforts to re-study and describe new materials (which have been developed in recent decades, e.g. [6,8,23,108–111]) should clarify this issue.

We recovered the Spinosauridae clade based on several synapomorphies (figure 31), among them the posterolateral orientation of the long axis of the medial condyle of the femur in distal view (314[1]; figure 23), representing an unambiguous synapomorphy. Internally, *Baryonyx* represents the first species to diverge, representing the outgroup of a larger clade containing *Ichthyovenator*, *Suchomimus* and the more deeply nested clade Spinosaurinae; that larger clade has a marginal pubic symphysis morphology (286[0]) as a synapomorphy. *Ichthyovenator* in turn is the outgroup of a smaller clade containing *Suchomimus* and Spinosaurinae, being supported by two synapomorphies of the locomotor apparatus: lateral wall of brevis fossa of the ilium in relation to the medial wall taller along whole length (265[0]; figure 4) and length to width ratio of the pubic peduncle less than or equal to 1 (272[0]). Finally, the Spinosaurinae clade presents two unambiguous synapomorphies (figure 31) related to dentition; however, there is no internal resolution for this clade. The dichotomy between Baryonychinae and Spinosaurinae has been recently questioned [53,54]; however, most of the recent phylogenetic approaches, even with low support, recover Baryonychinae as a natural group (e.g. [8,19,20,55,57]; see a summary in [112]). Our results do not recover a monophyletic Baryonychinae (figure 1), but rather taxa considered ‘Baryonychinae’ in a succession of outgroups of Spinosaurinae, as proposed by Sales & Schultz [54]. However, it is noteworthy that other species that are not based on the appendicular skeleton, or that are only poorly preserved, were not considered here (e.g. [19–21]), so more integrative approaches combined with new discoveries and revisions (e.g. [53] for *Baryonyx*) can shed light on this subject.

### 4.2. Locomotor apparatus morphology and main morphological changes throughout theropod evolution

One of the most remarkable and widespread features in dinosauromorphs and early dinosaurs is bipedalism [113,114]. Historically, this mode of locomotion is associated with using the forelimbs for food manipulation, and the hindlimbs in cursorial specializations; important in the origin of dinosaurs [115], considering that the retention of bipedalism occurred in several herbivorous species [114]. As such, obligate bipedalism and an erect hindlimb posture with slightly flexed hip and knee joints are ancestral features for dinosaurs, and may have been a key factor in the initial radiation of the clade during the Late Triassic and Early Jurassic [2,85,113].

However, throughout the Mesozoic, several dinosaur lineages acquired anatomical and functional modifications of the musculoskeletal structure of the pelvic girdle and hindlimbs, associated with modifications to their limb orientation, centre of mass, muscle sizes and leverages, and morphology/positions of origins and insertions of muscles. These modifications presumably enabled diversification of locomotor modes in several theropod lineages throughout their evolution [47–50,69–71,81,83,85,116–118]. Nevertheless, all known theropod dinosaurs retain obligate bipedalism, and unlike quadrupedalism, this acquisition probably had a unique origin before or at Dinosauria [2,46,85,114].

Despite this ancestral bipedalism, hypotheses of quadrupedalism in theropod species have been raised in some more recent literature. Based on characteristics such as the robustness of the humerus and elongation of the cranium and neck of the spinosaurid *Baryonyx walkeri* Charig & Milner [23] from the Early Cretaceous of Europe, Charig & Milner [30] proposed that this theropod dinosaur represented a facultative quadruped. However, Ibrahim *et al*. [15] commented that the morphology of another species closely related to *Baryonyx*, *Suchomimus tenerensis* Sereno *et al*. [11] from the Early Cretaceous of Africa, which has a better-preserved skeleton, does not support the hypothesis raised by Charig & Milner [30] concerning the Baryonychinae clade. In the same work, Ibrahim *et al*. [15] described a new specimen of the enigmatic, gigantic *Sp. aegyptiacus* Stromer [52] from the first part of the Upper Cretaceous (*ca* 95 Ma) of Africa. Based on some morphological features; such as the articulation between the sacral vertebrae and the ilium, and a short femur, in addition to the position of the estimated body's centre of mass; they hypothesized that this was the first (or only) theropod dinosaur which was an obligate quadruped on land. Ibrahim *et al*. [31] and Fabbri *et al*. [32] did not further address this issue in their description of new material along with biomechanical and morphological analyses more focused on the potential swimming abilities of *Spinosaurus*. However, Sereno *et al*. [16] produced a new three-dimensional computer model of the centre of mass of *Spinosaurus*, estimating that it was closer to the hips than Ibrahim *et al*. [15] reconstructed, and thus would have enabled normal bipedalism. Further integration of the anatomical insights for megalosauroids provided here could help resolve these controversies about locomotor function in spinosaurids.

Functional aspects related to bipedalism and gait gradually changed over macroevolutionary time in the lineage of theropod dinosaurs, which consequently gave rise to the most diverse locomotor mechanisms observed in birds [46,76,83–85,88]. However, such morphofunctional adaptations present a continuous series, or stepwise functional evolution [47,49,88]. As an example of this ‘gradual evolution’ in the avian lineage, features such as hip flexion and knee articulation, among others, stand out [49,76]. Early diverging tetanurans, as noted by Carrano *et al*. [6], represent a prime example of this transition from an ancestral dinosaurian locomotor morphology to a derived or ‘bird-like’ morphology present in coelurosaurs. Many morphological acquisitions related to the evolution of the locomotor system in dinosaurs occurred in parallel more than once throughout the evolution of the clade [83], among them more expanded iliac processes, changes of the morphology of the head of the femur and the lesser trochanter, many of which contributed to altered biomechanical functions in locomotion [47–49,83,84,88].

Carrano *et al*. [6] highlighted that even though there were variations in the locomotor morphology of early tetanurans throughout their evolution, such characteristics seem to have occurred to a lesser extent when compared with other theropod clades (e.g. ceratosaurs and coelurosaurs). Thus, early tetanurans had a relatively generalized locomotor morphology for early theropods. However, morphological variations, especially in megalosauroids (summarized in §3.4), suggest distinctions in functions of parts of the locomotor apparatus.

Based on our analysis, we summarize the following evolutionary aspects and their potential morphofunctional implications in megalosauroids:

#### 4.2.1. Pelvis

A low swollen ridge on the lateral surface of the ilium is present in Piatnitzkysauridae and other species such as *Afrovenator*, *Megalosaurus* and *Suchomimus* (figure 2); this ridge potentially indicates a strong separation between the origins of the ITC/IFE and ILFB muscles (e.g. [50]). The brevis fossa with a posterior width greater than the anterior one is the condition in *Marshosaurus*, *Eustreptospondylus* and spinosaurids (except *Ichthyovenator*) (figure 3); furthermore, in the spinosaurids *Suchomimus* and *Spinosaurus* the height of the lateral wall of the brevis fossa relative to the medial one is taller along the whole length (differing from other megalosauroids) (figure 4). Both latter conditions of the brevis fossa suggest a greater size of the CFB muscle (with the posterior enlargement) and might indicate a more restricted CFB origin and smaller muscle size anteriorly in *Suchomimus* and *Spinosaurus*. In general, the pubic peduncle length to width ratio in megalosauroids is between 1.3 and 1.75; however, in *Eustreptospondylus* and the spinosaurids *Suchomimus* and *Spinosaurus*, the plesiomorphic condition of the ratio less than or equal to 1 may indicate a more restricted origin of PIFI1 in these taxa. The presence of a ridge on the medial surface of the ilium adjacent to the preacetabular notch is a feature noted in *Ichthyovenator*; this condition combined with a larger peduncle may suggest an expanded PIFI1 origin. The posterior margin of the postacetabular blade of the ilium in megalosauroids is generally convex; however, in *Eustreptospondylus*, *Megalosaurus* and *Torvosaurus* (figure 11) the presence of a prominent posterodorsal process may indicate a greater extent of the origin of IT3 and somewhat a more restricted origin of the IFE at the posterior margin of the ilium.

The pubic shaft in almost all megalosauroids studied is straight, the exceptions being *Marshosaurus* with a ventrally concave shaft and *Spinosaurus* with a dorsally concave pubic shaft (figure 12). Such differences may not influence the area of origin or the size of the PIFE1–2 muscles; however, the moment arms of the associated musculature might have had at least slight changes with this disparate morphology. The shape of the pubic symphysis in non-spinosaurid megalosauroids is broad, whereas in spinosaurids (except *Baryonyx*) the morphology of the symphysis is marginal. This feature associated with other pelvic fusion conditions might be related to increased rigidity/strength of this structure, and the pubic boot may have provided stronger resistance to supporting body weight during sitting (as well as enlarged abdominal muscle insertions and improved inspiratory flow, as previously suggested (e.g. [86])). In spinosaurids (based on *Ichthyovenator* and *Suchomimus*), there is an increase in the size of the obturator foramen, which may reflect the reduction of the pelvic surface area and hence reduction of the associated musculature size (e.g. PIFE3) or even losses of muscles (e.g. parts of the *flexor cruris* ventral group).

The relative size of the ischium to the pubis in megalosauroids generally ranges from 75% to 80% (although this relationship is poorly understood in megalosaurids). A relatively larger ischium, greater than 80% of the pubis, is characteristic of *Torvosaurus* and *Spinosaurus*; such taxa may have had an increased area of origin of the associated musculature (e.g. PIFE3, ADD2, ISTR). The shaft of the ischium in piatnitzkysaurids and spinosaurids is straight, whereas in megalosaurids (except *Torvosaurus*) the shaft is ventrally curved (figure 14); these shape differences would at least slightly alter the moment arms (e.g. [84]) of muscles with ischial origins. Most megalosauroids have a reduced ischial antitrochanter, except for the spinosaurid *Ichthyovenator*, which has a large and notched antitrochanter. The latter feature may have limited abduction of the hindlimb as well as perhaps reduce stresses, similar to how the antitrochanter is assumed to function in Aves [119]; however, more quantitative, biomechanical research is needed in this regard, as antitrochanter function remains obscure. In the region ventral to the obturator process, a notch is present in piatnitzkysaurids, *Afrovenator*, and spinosaurids (except *Ichthyovenator*) (figure 15), suggesting that in these taxa the PIFE3 and ADD1 origins may have been reduced in size. The opposite is evident for the expanded apron morphology of the ischial symphysis observed in *Marshosaurus*, *Eustreptospondylus*, *Megalosaurus*, *Torvosaurus* and *Ichthyovenator*; which could be correlated with an enlargement of muscle origins, such as for ADD1 and PIFE3.

#### 4.2.2. Appendage

In the proximal part of the femur, the groove for the oblique ligament in the posterior region of the head is shallow in *Afrovenator*, *Megalosaurus*, *Torvosaurus* and *Spinosaurus*, which differs from the deep groove noted in other theropods. However, it is not clear whether this ligament and groove depth provided any special constraints to mobility, being a feature that is variable in Dinosauriformes [96]. In megalosauroids, the presence of an accessory trochanter that derives from the lesser trochanter in general represents a weak structure that forms only a slightly thickened margin. However, in *Suchomimus* the accessory trochanter is represented by a triangular flange (figure 20), and this structure is associated with the insertion of PIFI2, which suggests at least slight alterations in PIFI2 muscle actions in this species. On the distal part of the femur, the tibiofibularis crest in piatnitzkysaurids is broad, but in other megalosauroids this crest is narrow and longitudinal (figure 22); it is not clear how these differences might have altered the biomechanics of the knee joint. Two other features of the distal femur that may have altered locomotor biomechanics are the orientation of the medial condyle axis, which is posterolateral only in spinosaurids (figure 23); and the distal projection of the lateral and medial condyles, which is approximately equal in several megalosauroids, with a distal projection of the lateral condyle in *Leshansaurus*, and distal projection of the medial condyle in *Suchomimus* and *Spinosaurus* (figure 24), but further biomechanical studies are needed to unravel any implications of these structures. Finally, a shallow fibular fossa is the main feature of megalosauroids; except piatnitzkysaurids, which have a deep medial fossa on the fibula; this fossa might be a more concentrated origin of part of the digital flexor's muscles, or part of the ‘popliteus’/interosseous cruris/pronator profundus, which suggests greater robustness of this musculature in piatnitzkysaurids.

Together, these possible changes of muscle positions and sizes (reductions and expansions), and alterations of joint morphology, hint at widespread, complex changes of musculoskeletal function across Megalosauroidea. As these features sometimes are subtle or simply qualitatively described here, they deserve more rigorous quantitative characterization. Although there has been progress in studies of the evolution of the locomotor system in theropods (e.g. [49,50,69–71,81,84,85,118]), focus on groups such as the Megalosauroidea has been almost non-existent. Armed with the basic insights on morphological evolution presented here, future studies could, for example, map the appendicular musculature in Megalosauroidea and use such data to conduct quantitative biomechanical analyses of the functional impact of morphological traits (e.g. [69–71,94]), contextualizing Megalosauroidea with other theropods.

### 4.3. Morphological disparity of the locomotor apparatus in early theropods (especially Megalosauroidea)

Based on our disparity analyses focusing in megalosauroids, the greatest disparity calculated for the ilium is among megalosaurids; for the pubis it is in both piatnitzkysaurids and spinosaurids; for the ischium it is in spinosaurids; there is a clear distinction in femoral morphospace distribution for megalosauroids and other theropods; and piatnitzkysaurids show the greatest disparity of zeugopodium characters (figures 29 and 30). Based on our interpretations, the most homoplastic structures are: (i) the ilium in Orionides; (ii) the pubis, to a lesser extent, among non-carcharodontosaur averostrans; (iii) the ischium among most species; and (iv) tibia/fibula, to a lesser extent, between non-spinosaurid megalosauroids and neovenatorids.

Using morphological data and taxa different from those used here, an approach carried out by Novas *et al*. [67] evaluated key aspects of morphological disparity in theropod dinosaurs. Considering all morphological characters of the skeleton, Novas *et al*. [67] found that the clade that occupies the largest area in morphospace is Coelurosauria, which is expected given the wide variety of forms found in this clade; including Aves [4,67]. Similarly, Brusatte *et al*. [3] found, based on cranial morphology and palaeoecology, that non-carnivorous theropods have greater morphological disparity than carnivorous theropods; in a second taxonomically based approach, they showed that the greatest disparities are in ceratosaurs and in coelurosaurian oviraptorosaurs, interestingly noting that non-carnivorous taxa are included in both clades. Both studies [3,67] suggest that theropod skulls have broad disparity, probably relating to disparate feeding mechanisms and ecologies.

Considering postcranial elements, Novas *et al*. [67] divided their analyses into several structures. They obtained the following results for the locomotor apparatus: (i) pelvic girdle: three distinct morphospaces, occupied by non-neotheropods, non-megalosauroids tetanurans and coelurosaurs; a large area occupied by coelurosaurs could in part be explained by the retroverted pubis, absence of the supraacetabular crest and the presence of a transversely enlarged ischial peduncle noted in some taxa; and (ii) hindlimb stylopodium and zeugopodium: four distinct morphospaces, occupied by non-neotheropods, non-averostrans, ceratosaurs and tetanurans. Again, coelurosaurs occupied a large area, especially with the influence of the greatest variance component (i.e. PCoA1).

In our complete analysis (figure 29*a*), the distribution of megalosaurids in the morphospace can be explained by the puboischiadic plate, morphology of the femoral condyles and the morphology/development of the fibular crest of the tibia. Considering the pubis (figure 29*e*), megalosaurids occupy a large area in morphospace influenced by PCO2, which can be explained by both proximal and distal pubic articulations. In the analysis of the ischium (figure 30*a*), the distribution of megalosaurids, influenced by PCO1, seems to be explained by the relative length of the pubis/ischium, the orientation of the ischial shaft and the ventral notch of the obturator process. Analysing only the femur (figure 30*c*), a clear distinction among Orionides and non-Orionides theropods is noted. Considering the zeugopodium (figure 30*e*), the great overlap area is shared among the megalosauroid piatnitzkysaurids, megalosaurids and the allosauroid metriacanthosaurids. The distribution of the megalosaurids is influenced by the morphology of the fibular crest of the tibia. As final remarks, throughout the macroevolution of tetanuran theropod dinosaurs, the ilium, ischium and the femur seem to play a role in the differentiation of several taxa, appearing to be the most disparate structures of the locomotor system. Meanwhile, the femur in megalosauroids causes the group to be segregated in the morphospace between non-Orionides and Avetheropods. New approaches revising the characters used here and complementing them with autapomorphic proposals for some species (e.g. [15]) may increase our understanding of this dinosaur clade.

## Data Availability

The data are provided in electronic supplementary material [68].

## Appendix A

Clades/species list: SAUROPODOMORPHA; *Eoraptor lunensis*, THEROPODA; Herrerasauridae, *Herrerasaurus ischigualastensis*, NEOTHEROPODA; Coelophysoidea, *Dilophosaurus wetherilli*, Coelophysidae, *Coelophysis bauri*, *Co*. *rhodesiensis*, AVEROSTRA; Ceratosauria, *Elaphrosaurus bambergi*, *Ceratosaurus nasicornis*, Abelisauroidea, *Masiakasaurus knopleri*, *Majungasaurus crenatissimus*, TETANURAE; *Sinosaurus sinensis*, *Cryolophosaurus ellioti*, *Monolophosaurus jiangi*, *Chuandongocoelurus primitivus*, ORIONIDES; Megalosauroidea, Piatnitzkysauridae, *Condorraptor currumili*, *Marshosaurus bicentesimus*, *Piatnitzkysaurus floresi*, Megalosauria, *Eustreptospondylus oxoniensis*, Spinosauridae, *Angaturama limai*, *Baryonyx walkeri*, *Ichthyovenator laosensis*, *Irritator challengeri*, *Spinosaurus aegyptiacus*, *Suchomimus tenerensis*, Megalosauridae, *Afrovenator abakensis*, *Dubreuillosaurus valesdunensis*, *Leshansaurus qianweiensis*, *Magnosaurus nethercombensis*, *Piveteasaurus divesensis*, *Duriavenator hesperis*, *Megalosaurus bucklandii*, *Torvosaurus tanneri*, AVETHEROPODA; Allosauroidea, Metriacanthosauridae, *Yangchuanosaurus shangyouensis*, *Y*. *zigongensis*, *Metriacanthosaurus parkeri*, *Shidaisaurus jinae*, *Siamotyrannus isanensis*, *Sinraptor hepingensis*, *Si*. *dongi*, Allosauria, Allosauridae, *Allosaurus europaeus*, *Al*. *fragilis*, *Al*. *jimmadseni*, *Saurophaganax maximus*, Carcharodontosauria, Neovenatoridae, *Neovenator salerii*, *Chilantaisaurus tashuikouensis*, Megaraptora, *Aerosteon riocoloradensis*, *Australovenator wintonensis*, *Fukuiaraptor kitadaniensis*, *Megaraptor namunhuaiquii*, Carcharodontosauridae, *Acrocanthosaurus atokensis*, *Concavenator corcovatus*, *Eocarcharia dinops*, *Shaochilong maortuensis*, *Carcharodontosaurus iguidensis*, *Ca*. *saharicus*, *Giganotosaurus carolinii*, *Mapusaurus roseae*, *Tyrannotitan chubutensis*, COELUROSAURIA; *Lourinhanosaurus antunesi*, *Compsogntahus longipes*, *Ornitholestes hermanni*, *Proceratosaurus bradleyi*.

## Appendix B

The entire morphological character list can be accessed in Carrano *et al*. [6], here is presented the list of characters scored/mapped in this work:

261. Pelvic elements, articulations in adults: separate (0), fused (1).

262. Ilium, large external pneumatic foramina and internal spaces: absent (0), present (1).

263. Ilium, vertical ridge on lateral surface of blade dorsal to acetabulum: absent (0), low swollen ridge (1), low double ridge (2).

264. Ilium, posterior width of brevis fossa: subequal to anterior width, fossa margins subparallel (0), twice anterior width, fossa widens posteriorly (1).

265. Ilium, height of lateral wall of brevis fossa relative to medial wall: taller along whole length (0), shorter anteriorly, exposing medial wall in lateral view (1).

266. Ilium, morphology between supraacetabular crest and brevis shelf on lateral surface: gap (0), continuous ridge (1).

267. Ilium, ventrolateral development of supraacetabular crest: large/pendant ‘hood’ (0), reduced shelf (1).

268. Ilium, orientation of pubic peduncle: mostly ventral (0), mostly anterior or ‘kinked’ double facet with anterior and ventral components (1).

269. Ilium, shape of acetabular margin of pubic peduncle: transversely convex or flat (0); transversely concave (1).

270. Ilium, relative sizes of pubic and ischial articulations: subequal (0), pubic articulation greater than or equal to 130% of iliac articulation (1).

271. Ilium, morphology of ischial peduncle: rounded (0), acuminate (1).

272. Ilium, pubic peduncle length to width ratio: less than or equal to 1 (0), 1.3–1.75 (1), greater than 2 (2). Ordered.

273. Ilium, ridge on medial surface adjacent to preacetabular notch: absent (0), present (1), strongly developed, forming a shelf (2). Ordered.

274. Ilium, preacetabulum length relative to anterior edge of pubic peduncle: reaches anteriorly to same point as (brachyiliac) (0), or well past (dolichoiliac) (1).

275. Ilium, depth of preacetabular process: shallow (0), deep (1).

276. Ilium, anteroventral lobe of preacetabular process: absent (0), present (1).

277. Ilium, shape of dorsal margin: convex (0), straight (1).

278. Ilium, postacetabulum length relative to ischial peduncle length: less than or equal to (0), greater than (1).

279. Ilium, depth of postacetabular process: shallow (0), deep (1).

280. Ilium, shape of posterior margin of postacetabular process: convex (0), concave (1), straight (2), with prominent posterodorsal process but lacking posteroventral process (3).

281. Puboischiadic plate, morphology and foramina/notches: fully closed along midline, 3 fenestrae (0), open along midline, 1 fenestra (obturator foramen of pubis) and 1–2 notches (1), open along midline, 0 fenestrae, 1–2 notches (2).

282. Pubis, shaft orientation: straight (0), ventrally concave (1); dorsally concave (2).

283. Pubis, articulation between apices in adults: unfused (0); fused (1).

284. Pubis, contact between distal portions: separate distally (0), contacting (1), contacting with slit-like opening proximal to distal expansion (interpubic fenestra) (2).

285. Pubis, angle between long axes of shaft and boot: 75–90° (0), less than 60° (1).

286. Pubis, morphology of symphysis: marginal (0), broad (1).

287. Pubis, morphology of obturator foramen: small and subcircular (0), large and oval (1).

288. Pubis, anterior expansion of distal end: absent (0), present (1).

289. Pubis, boot length relative to shaft length: less than (0), greater than 30% (1), greater than 60% (2). Ordered.

290. Pubis, shape of boot in ventral view: broadly triangular (0), narrow, with subparallel margins (1).

291. Pubis, articulation with ilium: planoconcave (0), peg-and-socket (1).

292. Ischium, length relative to pubis length: 75–80% (0), less than or equal to 70% (1), greater than 80% (2).

293. Ischium, shaft orientation: straight (0), ventrally curved (1).

294. Ischium, articulation with ilium: planoconcave (0), peg-and-socket (1).

295. Ischium, morphology of antitrochanter: large and notched (0), reduced (1).

296. Ischium, notch ventral to obturator process: absent (0), present (1).

297. Ischium, morphology of symphysis: unexpanded (0), expanded as apron (1).

298. Ischium, cross-sectional shape of paired midshafts: oval (0), heart-shaped, medial portions of shafts extend posteriorly as midline flange (1).

299. Ischium, morphology of distal end: rounded (0), expanded, triangular (1).

300. Ischium, articulation at distal end in adults: separate (0), fused (1).

301. Femur, head orientation: 45° anteromedial (0), 10–30° anteromedial (1), medial (2). Ordered.

302. Femur, head angle: ventromedial (0), horizontal (medial) (1), dorsomedial (2). Ordered.

303. Femur, groove on proximal surface of head-oriented oblique to long axis of head (‘articular groove’ or fovea capitis): absent (0), present (1).

304. Femur, oblique ligament groove on posterior surface of head: shallow, groove bounding lip does not extend past posterior surface of head (0), deep, bound medially by well-developed posterior lip (1).

305. Femur, placement of lesser trochanter relative to femoral head: does not reach ventral margin (0), rises past ventral margin (1), rises to proximal surface (2). Ordered.

306. Femur, morphology of anterolateral muscle attachments at proximal end: continuous trochanteric shelf (0), distinct lesser trochanter and attachment bulge (1).

307. Femur, development of fourth trochanter: prominent semioval flange (0), very weak or absent (1).

308. Femur, distinctly projecting accessory trochanter (derived from lesser trochanter): weak, forms slightly thickened margin of lesser trochanter (0), present as triangular flange (1).

309. Femur, *M*. *femorotibialis externus* origin medially on anterodistal surface: faint, small rugose patch (0), pronounced rugose depression that extends to distal femur (1).

310. Femur, development of medial epicondyle: rounded (0), ridge (1).

311. Femur, distal extensor groove: absent (0), present (1).

312. Femur, morphology and orientation of tibiofibularis crest: broad (0), narrow, longitudinal (1), lobular, oblique (2).

313. Femur, infrapopliteal ridge connecting medial distal condyle and crista tibiofibularis: absent (0), present (1).

314. Femur, orientation of long axis of medial condyle in distal view: anteroposterior (0), posterolateral (1).

315. Femur, projection of lateral and medial distal condyles: approximately equal (0), lateral projects distinctly further than medial, distal surface of medial is gently flattened (1), medial projects distinctly further than lateral (2).

316. Femur, morphology of distal end: central depression connected to crista tibiofibularis by a narrow groove (0), anteroposteriorly oriented shallow trough separating medial and lateral convexities (1).

317. Tibia, lateral malleolus: backs astragalus (0), overlaps calcaneum (1).

318. Tibia, shape of edge of lateral malleolus: smoothly curved (0), tabular notch (1).

319. Tibia, morphology of distal cnemial process: rounded (0), expanded proximodistally (1).

320. Tibia, morphology of lateral (fibular) condyle: large (0), small and lobular (1).

321. Tibia, anterolateral process of lateral condyle: absent or horizontal projection (0), prominent, curves ventrally (1).

322. Tibia, anteromedial buttress for astragalus: absent (0), ventral (1), marked oblique step-like ridge (2), reduced oblique ridge (3), bluntly rounded vertical ridge on medial side (4).

323. Tibia, morphology of fibular crest: narrow (0), bulbous (1).

324. Tibia, development of fibular crest: extends to proximal end of tibia as high crest (0), extends to proximal end of tibia as low ridge (1), does not extend to proximal end of tibia (2). Ordered.

325. Fibula, depth of fibular fossa on medial aspect: groove (0), shallow fossa (1), deep fossa (2).

326. Fibula, position of fibular fossa on medial aspect: posterior edge (0), central (1).

327. Fibula, size of iliofibularis tubercle: faint scar (0), large (1), anterolaterally curving flange (2).

328. Fibula, size of proximal end relative to width of proximal tibia: less than 75% (0), greater than or equal to 75% (1).

## Appendix C

Remarks on character/scoring:

264. Ilium, posterior width of brevis fossa: subequal to anterior width, fossa margins subparallel (0), twice anterior width, fossa widens posteriorly (1).

Remarks: State ‘?’ in *Piatnitzkysaurus* [6] modified to state ‘0’. State ‘?’ in *Ichthyovenator* [57] modified to state ‘1’.

265. Ilium, height of lateral wall of brevis fossa relative to medial wall: taller along whole length (0), shorter anteriorly, exposing medial wall in lateral view (1).

Remarks: State ‘?’ in *Suchomimus* [6] modified to state ‘0’. State ‘?’ in *Ichthyovenator* [57] modified to ‘1’.

271. Ilium, morphology of ischial peduncle: rounded (0), acuminate (1).

Remarks: State ‘1’ in *Ichthyovenator* [57] modified to ‘0’.

277. Ilium, shape of dorsal margin: convex (0), straight (1).

Remarks: State ‘1’ in *Ichthyovenator* [57] modified to ‘0’.

278. Ilium, postacetabulum length relative to ischial peduncle length: less than or equal to (0), greater than (1), 2x (2).

Remarks: State '2' removed.

282. Pubis, shaft orientation: straight (0), ventrally concave (1); dorsally concave (2).

Remarks: State '1' changed from ‘ventrally curved’ [6] to ‘ventrally concave’; state '2' dorsally concave added.

286. Pubis, morphology of symphysis: marginal (0), broad (1).

Remarks: State ‘1’ in *Ichthyovenator* [57] modified to ‘0’.

287. Pubis, morphology of obturator foramen: small and subcircular (0), large and oval (1).

Remarks: State ‘1’ in *Ichthyovenator* [57] modified to ‘0’.

290. Pubis, shape of boot in ventral view: broadly triangular (0), narrow, with subparallel margins (1).

Remarks: State ‘?’ in *Suchomimus* [6] modified to state ‘0’.

297. Ischium, morphology of symphysis: unexpanded (0), expanded as apron (1).

Remarks: State ‘?’ in *Suchomimus* [6] modified to state ‘0’.

299. Ischium, morphology of distal end: rounded (0), expanded, triangular (1).

Remarks: State ‘?’ in *Ichthyovenator* [57] modified to ‘0’.

303. Femur, groove on proximal surface of head-oriented oblique to long axis of head (‘articular groove’ or fovea capitis): absent (0), present (1).

Remarks: State ‘?’ in *Baryonyx* and *Piatnitzkysaurus* [6] modified to state ‘1’; inclusion of fovea capitis as the character description, since this groove refers to the pubofemoral and ischiofemoral ligaments that form the ligamentum captis femoris, which is inserted onto the fovea capitis on the proximal femoral head (see [96]).

304. Femur, oblique ligament groove on posterior surface of head: shallow, groove bounding lip does not extend past posterior surface of head (0), deep, bound medially by well-developed posterior lip (1).

Remarks: State ‘?’ in *Suchomimus* [6] modified to state ‘1’.

306. Femur, morphology of anterolateral muscle attachments at proximal end: continuous trochanteric shelf (0), distinct lesser trochanter and attachment bulge (1).

Remarks: State ‘1’ in *Irritator* [6] modified to state ‘?’.

315. Femur, projection of lateral and medial distal condyles: approximately equal (0), lateral projects distinctly further than medial, distal surface of medial is gently flattened (1), medial projects distinctly further than lateral (2).

Remarks: State '2' medial projects distinctly further than lateral added; state ‘0’ in *Suchomimus* [6] modified to state ‘2’.

320. Tibia, morphology of lateral (fibular) condyle: large (0), small and lobular (1).

Remarks: State ‘?’ in *Suchomimus* [6] modified to state ‘1’.

321. Tibia, anterolateral process of lateral condyle: absent or horizontal projection (0), prominent, curves ventrally (1).

Remarks: State ‘?’ in *Suchomimus* [6] modified to state ‘0’.

## Appendix D

List of ordered characters' (following [57]): 2, 4, 6, 12, 24, 47, 74, 96, 129, 150, 152–153, 156, 172, 180, 189, 193, 197, 223, 231, 233, 249, 272–273, 281, 289, 301–302, 305, 324, 335 and 341.

## Appendix E

Taxa that do not have the studied pelvic and hindlimb elements preserved were removed from the disparity analyses. The taxa removed in analysis (i) and remaining analyses were *Angaturama*, *Duriavenator*, *Eocarcharia*, *Irritator*, *Piveteasaurus*, *Proceratosaurus* and *Shaochilong*. For the analysis (ii) we removed *Dubreuillosaurus* and *Megaraptor*. For the analysis (iii) we removed *Australovenator*, *Chilantaisaurus*, *Dubreuillosaurus* and *Fukuiraptor*. For the analysis (iv) we removed *Aerosteon*, *Australovenator*, *Chilantaisaurus*, *Chuandongocoelurus*, *Dubreuillosaurus*, *Fukuiraptor*, *Magnosaurus*, *Megaraptor* and *Leshansaurus*. For the analysis (v) we removed *Aerosteon*, *Ichthyovenator*, *Megaraptor*, *Monolophosaurus*, *Shidaisaurus*, and *Siamotyrannus*. For the analysis (vi) we removed *Ichthyovenator*, *Megaraptor*, *Monolophosaurus*, *Shidaisaurus*, *Siamotyrannus* and *Si*. *hepingensis*.
